# Recent advances and perspectives of CeO_2_-based catalysts: Electronic properties and applications for energy storage and conversion

**DOI:** 10.3389/fchem.2022.1089708

**Published:** 2022-12-08

**Authors:** Xianwei Wang, Jingyi Wang, Yafei Sun, Kanghui Li, Tongxin Shang, Ying Wan

**Affiliations:** The Education Ministry Key Laboratory of Resource Chemistry, Joint International Research Laboratory of Resource Chemistry of Ministry of Education, Shanghai Key Laboratory of Rare Earth Functional Materials, Shanghai Frontiers Science Center of Biomimetic Catalysis, Shanghai Non-Carbon Energy Conversion and Utilization Institute, Shanghai Normal University, Shanghai, China

**Keywords:** cerium dioxide, catalysts, photocatalysis, electrocatalysis, energy storage and conversion, electronic properties

## Abstract

Cerium dioxide (CeO_2_, ceria) has long been regarded as one of the key materials in modern catalysis, both as a support and as a catalyst itself. Apart from its well-established use (three-way catalysts and diesel engines), CeO_2_ has been widely used as a cocatalyst/catalyst in energy conversion and storage applications. The importance stems from the oxygen storage capacity of ceria, which allows it to release oxygen under reducing conditions and to store oxygen by filling oxygen vacancies under oxidizing conditions. However, the nature of the Ce active site remains not well understood because the degree of participation of *f* electrons in catalytic reactions is not clear in the case of the heavy dependence of catalysis theory on localized *d* orbitals at the Fermi energy *E*_*F*_. This review focuses on the catalytic applications in energy conversion and storage of CeO_2_-based nanostructures and discusses the mechanisms for several typical catalytic reactions from the perspectives of electronic properties of CeO_2_-based nanostructures. Defect engineering is also summarized to better understand the relationship between catalytic performance and electronic properties. Finally, the challenges and prospects of designing high efficiency CeO_2_-based catalysts in energy storage and conversion have been emphasized.

## 1 Introduction

Nowadays, most of the energy demand (more than 80%) is met by fossil fuels (such as coal, oil, and natural gas). However, the rapidly growing energy consumption gives rise to serious environmental concerns and energy crisis (Xie et al., 2017). Non-conventional energy sources, such as solar, wind, hydropower, etc., are being considered as possible sources of energy to meet the growing demand and alleviate environmental destruction (Wang J. et al., 2019). It has been clear for decades that renewable energy sources play important role in the modern grid. While the intermittent nature of these renewable energy sources will lead to a significant mismatch between supply and demand. Electrical energy conversion and storage from different renewable energy sources is a high-efficiency and clean strategy that takes full advantage of all kinds of energy. Many new technologies for energy conversion and storage are under development, which is expected to meet the requirement of their practical applications. Especially, the development of the electrochemical and photochemical processes is a prospective goal to sustainably and cleanly realize the efficient conversion and storage of many energy molecules, including carbon dioxide and a series of C_2+_ hydrocarbons and oxygenates, hydrogen, sulfur, nitrogen, and so on (Li Q. et al., 2021). To realize this expectation, it is necessary and urgent to develop photo (electro) catalysts with high catalytic activity and improved selectivity towards the high-efficiency energy molecules transformations. Numerous catalysts have been developed for the energy molecules conversion reactions such as carbon dioxide reduction reaction (CO_2_RR), hydrogen evolution reaction (HER), oxygen evolution reaction (OER), sulfur reduction reaction (SRR), etc., (Cai et al., 2021). For those important reactions, various carbon-based, metal-based, and metal oxide-based catalysts have been widely investigated. However, their performances, such as activity, stability, cost, and so on, are still need to strengthen. Especially, the conversion efficiency and the selectivity of the developed photocatalysts are still far from satisfactory up to now. Therefore, the development of better catalysts with the necessary selectivity and efficiency for the relevant chemical reactions is urgent.

CeO_2_, a widely studied rare Earth oxide, has gained promising applications in photocatalysis and electrocatalytic energy storage and conversion (Montini et al., 2016). Cerium is the most abundant of the rare Earth elements accounting for around 0.0046 wt% of the Earth’s crust. CeO_2_, as the most common oxide of cerium element, has good stability with a cubic fluorite crystal structure. Specifically, each Ce^4+^ is coordinated with eight adjacent O^2–^ to form an octahedral interstitial, and each O^2–^ is coordinated with four adjacent Ce^4+^ to form a tetrahedral unit in the CeO_2_ unit cell (Li and Shen, 2014). CeO_2_ possesses unique electronic configurations of [Xe]4*f* ^1^5*d* ^1^6*s* ^2^ resulting in excellent physical and chemical properties, for example, the different colors for the CeO_2_ with different stoichiometry due to the charge transfer between Ce^4+^ and O^2–^ (Melchionna and Fornasiero, 2014). The energy of the inner 4*f* level is nearly the same as that of the outer or valence 5*d* and 6*s* levels, thus small amounts of energy can change the relative occupancy of these electronic levels and give rise to a variable electronic structure, which is the intrinsic property for CeO_2_ with application potentials in catalysis, energy conversion and storage, and other felids. For non-stoichiometric CeO_2–*x*_, four outer electrons of each cerium atom transfer to the two adjacent cerium atoms with the oxygen atom *via* the oxygen *p* orbital, which is beneficial to the reduction of Ce^4+^ to Ce^3+^ (Shea, 2020). Therefore, Ce^3+^ and Ce^4+^ are steadily exist and facilely switch between these two valence states, and the reversible conversion of the two valence state distributions of cerium ions ensures the formation or elimination of oxygen vacancies. The multivalence property of CeO_2_ is the key to achieve the enhanced performances in electrocatalytic and photocatalytic applications, as it benefits to generate strong interactions with reactants or other components in catalysts (Ganduglia-Pirovano et al., 2007; Gao et al., 2017; Li Q. et al., 2021). Besides, the reversible valence characteristics endow the CeO_2_ with a better catalytic performance by manipulating the oxygen vacancies concentration to build defect-rich structures (Kong et al., 2020).

**FIGURE 1:**
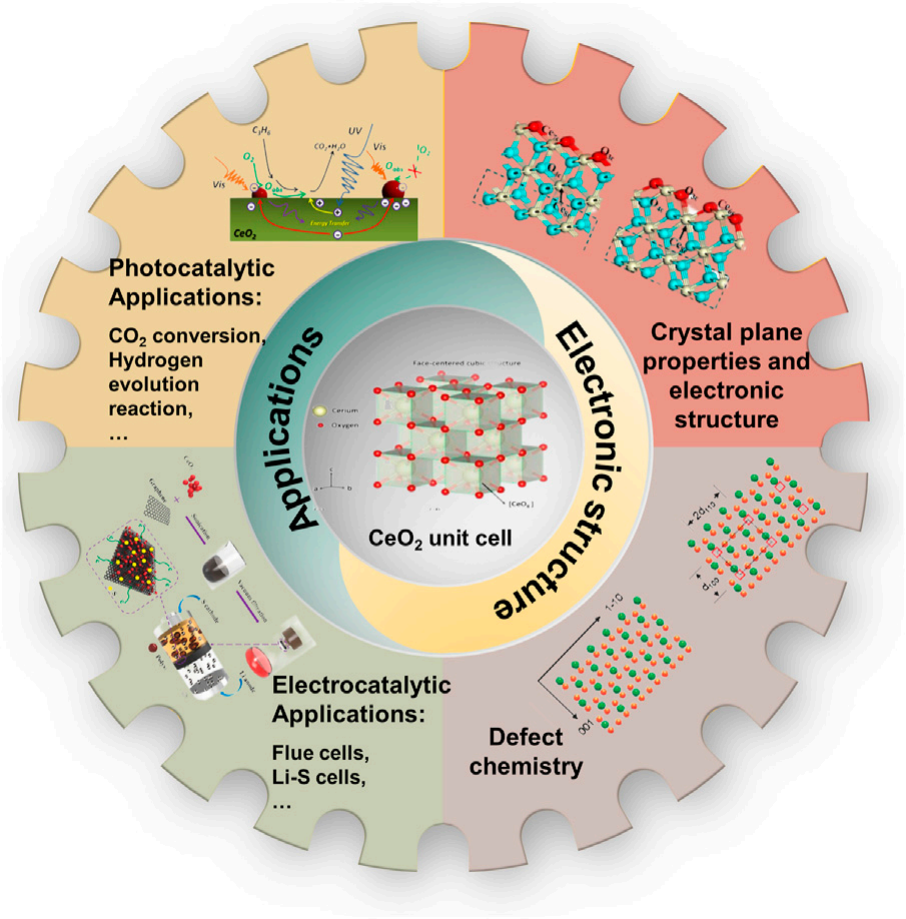
Illustration of electronic properties and catalytic applications of CeO_2_.

CeO_2_-based nanostructures have been widely reported. Previously, there are several reviews on the properties, characterizations, and applications of CeO_2_ (Ta et al., 2008; Wang H. et al., 2022; Kumaran et al., 2022). However, none of them have summarized the late advances on CeO_2_ from a perspective of understanding the relationship between electronic structures and catalytic application. Hence, a timely and focused progress report of CeO_2_ electronic properties is expected to further accelerate the development of CeO_2_-based emerging materials and promote their diverse applications. In this review, we summarize the recent development in the understanding and regulating strategy of electronic properties of CeO_2_-based nanostructures. The defects engineering is also summarized to better understand the relationship between catalytic performance and electronic properties. We then overview the catalytic applications of CeO_2_-based nanostructures in energy conversion and storage and discuss the mechanisms for several representative catalytic reactions and electrochemical cells in the presence of CeO_2_. Finally, the challenges and prospects of designing high efficiency CeO_2_-based catalysts in energy storage and conversion have been emphasized (Figure 1).

## 2 Electronic properties of CeO_2_-based nanostructures

### 2.1 Crystal plane properties and electronic structure

CeO_2_ has been widely used as the critical component (active site or support) and electronic promoter in heterogeneous catalysts. Further improving its selectivity and activity for certain reactions hold great promise by the materials engineering methods at the atomic level. It is also important to offer a more detailed understanding of the effects of various material design methods and the origin of the enhanced reactivity of these modified materials. For CeO_2_, the unique electronic configuration and the stable crystalline structure are the essential factors for its catalysis and electrochemical purpose.

**FIGURE 2:**
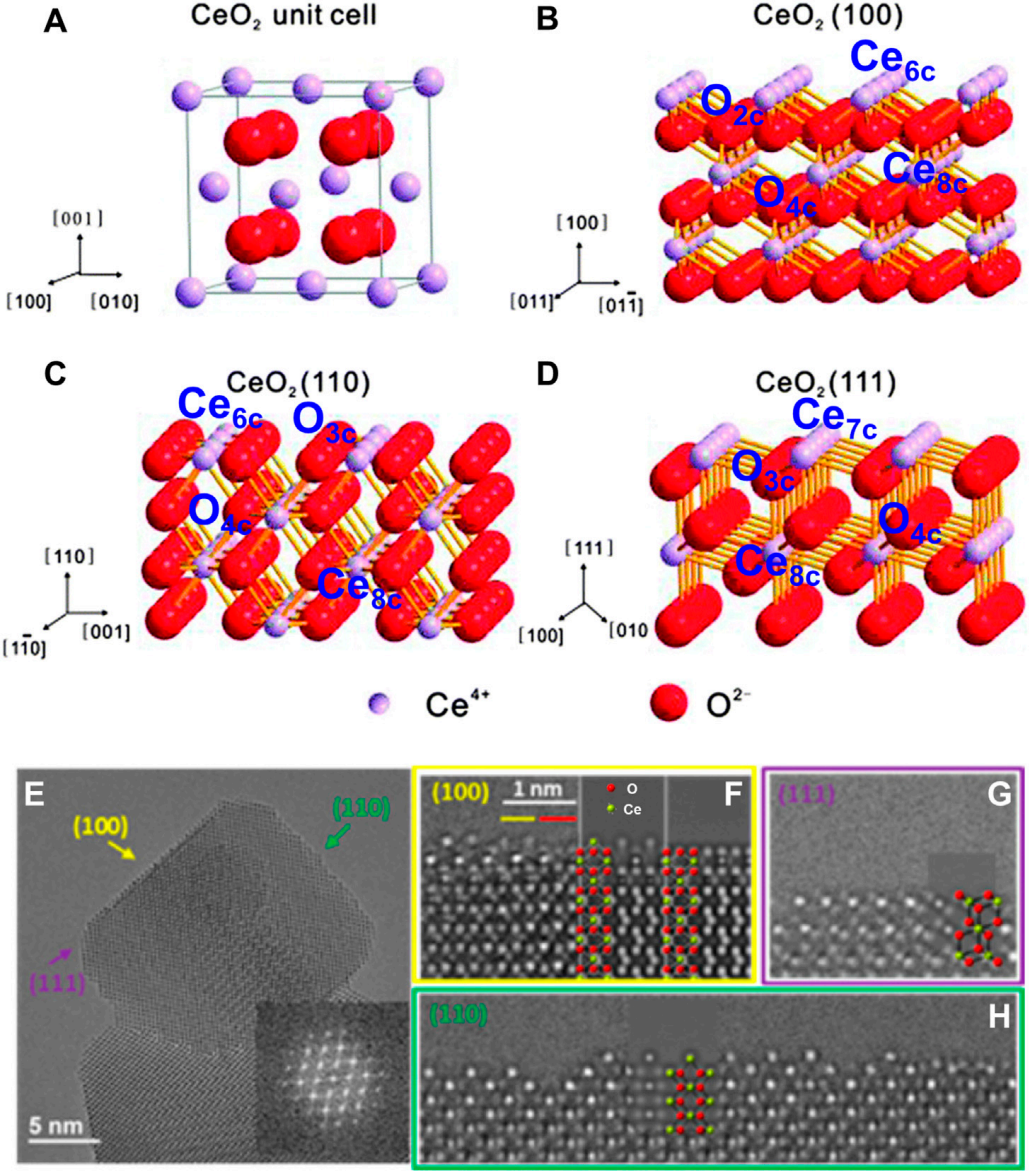
Atomic configurations and crystal facets of CeO_2_: **(A–D)** illustrations of the unit cell **(A)** and the (100), (110), and (111) facets with marked coordination number of cerium and oxygen atoms **(B–D)**. Reproduced with permission (Li and Shen, 2014). Copyright 2014, Royal Society of Chemistry. **(E–H)** The high-resolution electron microscopy (HREM) images and corresponding simulated HREM images of the three surfaces of a typical CeO_2_ nanocube. Reproduced with permission (Lin et al., 2014). Copyright 2013, American Chemical Society.

**FIGURE 3:**
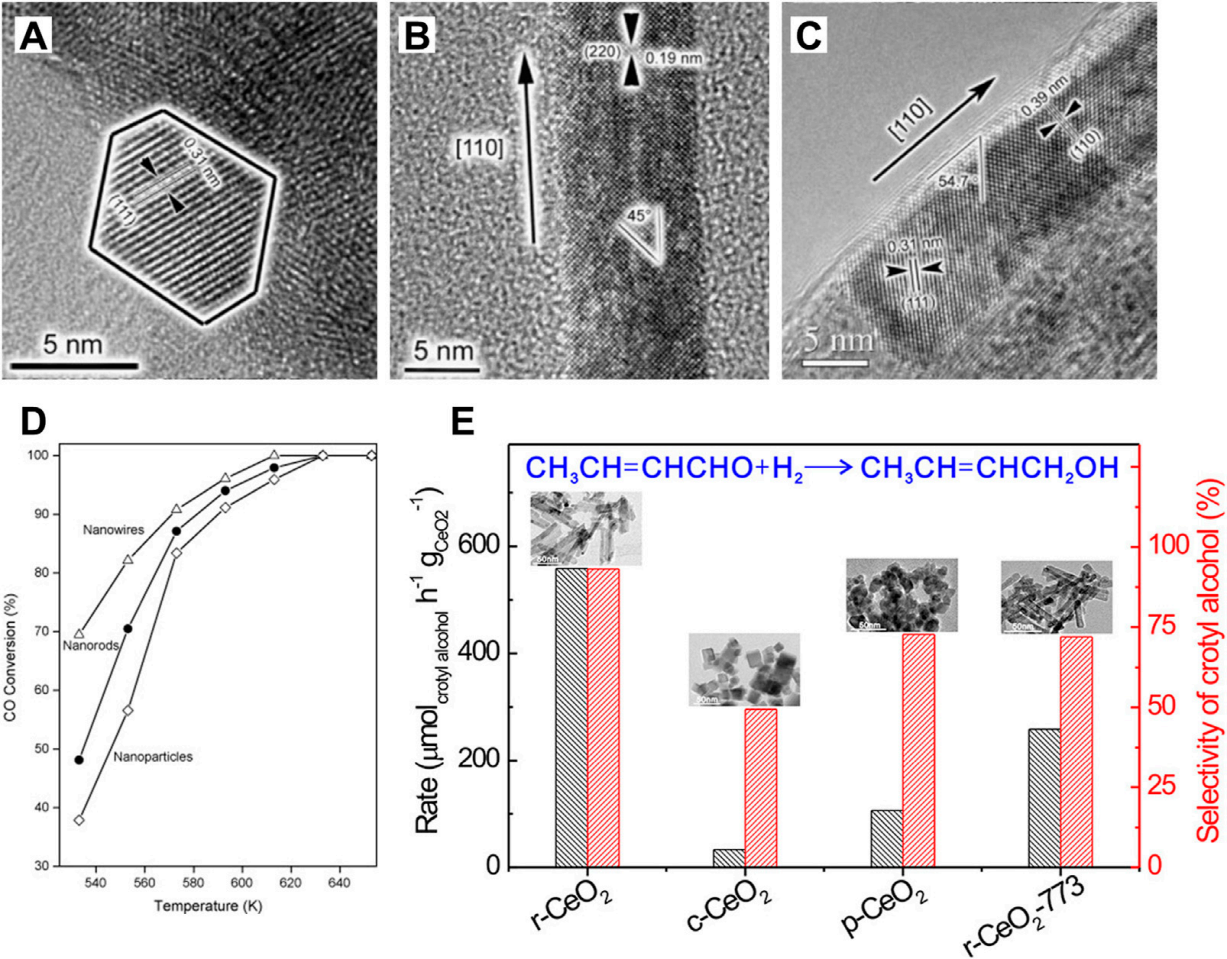
Crystal-plane-controlled catalytic performances: **(A–C)** Transmission electronic microscopy images of CeO_2_ nanoparticles **(A)**, nanorods **(B)** and nanowires **(C)**; **(D)** CO conversions over the CeO_2_ nanostructures with different morphologies. Reproduced with permission (Tana et al., 2009). Copyright 2009, Elsevier. **(E)** Formation rate and catalytic selectivity of crotyl alcohol for the gas-phase selective hydrogenation of crotonaldehyde catalyzed by various CeO_2_ at 323 K. Reproduced with permission (Zhang Z. et al., 2020). Copyright 2020, American Chemical Society.

CeO_2_ is an *n*-type semiconductor with a band gap of about 3.2 eV. CeO_2_ nanocrystal presents the fluorite crystal structure with space group *Fm*3*m* at the temperature range from room temperature to the melting point. As shown in Figure 2A, the fluorite structure consists of a face-centered cubic (f.c.c.) unit cell of cations with anions occupying the octahedral interstitial sites. In a CeO_2_ unit cell, each Ce^4+^ is coordinated with eight oxygen ions nearby, and each O^2−^ in the tetrahedral space coordinates with the four nearest Ce^4+^. Generally, CeO_2_ exposes three thermodynamically stable surfaces and the stability follows the order (100) < (110) < (111) according to the surface energy obtained from the density functional theory (DFT) calculations (Figures 2B–D). As shown in Figures 2E–H, the atomic structures of these exposed facets of CeO_2_ nanocubes have been observed and determined using aberration-corrected high-resolution electron microscopy by Lin et al. (2014). In comparison to the bulk CeO_2_ materials, the cerium and oxygen atoms are unsaturated in all three exposed facets with a lower coordination number and a higher activity. Specifically, the polar (100) surface is terminated by sixfold-coordinated cerium atoms (Ce_6c_) and twofold-coordinated oxygen atoms (O_2c_); the (110) surface is terminated by a CeO_2_ plane with sixfold cerium (Ce_6c_) and threefold oxygen atoms (O_3c_); and the (111) surface is terminated by sevenfold-coordinated cerium atoms (Ce_7c_) and threefold-coordinated oxygen atoms (O_3c_) (Spezzati et al., 2019; Zhong and Gong, 2019). The surfaces (111) and (110) have neutral charges, while (100) is made up of a series of charged planes and a dipole moment. The performances in various catalytic reactions of CeO_2_-based catalysts are greatly related to their exposed facets, because the chemical state of surface cerium ions and the concentration of oxygen vacancies, which can construct the solid frustrated Lewis pair sites and hence influence the adsorption/activation energy of reactants on the surface, are expected to vary with their hosted facets (Zhang et al., 2017; Zhang Z. et al., 2020; Zhu et al., 2020). Both theoretical and experimental studies have demonstrated that the (100) and (110) surfaces of CeO_2_ are more reducible and active than the (111) surface (Trovarelli and Llorca, 2017), which is accordance with the sequence of the vacancy-formation energies (111) > (100) > (110) [2.60 eV for (111) surface, 2.27 eV for (100) surface and 1.99 eV for (110) surface] (Nolan et al., 2005). Amoresi et al. (2019) have synthesized CeO_2_ nanocrystals with different exposed crystalline planes by the morphology controlling and found that the hexagon-shaped CeO_2_ with dominant (111), (110), and (311) crystal planes has the best photocatalytic efficiency and highest degradation rate of organic pollutants due to its largest band gap energy and the highest (110) and lowest (311) electron density. Shen and co-authors have also investigated the relationships between the morphologies of CeO_2_ nanowires, nanorods, and nanoparticles and their redox and catalytic performances (Tana et al., 2009). The most reactive planes, known as the active (100) and (110) planes, are found in CeO_2_ nanorods and nanowires, while the least reactive planes, known as the (111) planes, are located in CeO_2_ nanoparticles (Figures 3A–C). As expected, the CeO_2_ nanoparticles presented the lowest CO conversion at the low temperature range (Figure 3D). The CeO_2_ nanowires dominated by the reactive (110) and (100) planes benefited to expose a large proportion of active planes on the surface, which resulted in a much higher activity for CO oxidation. Besides, Zhang Z. et al. (2020) have demonstrated that the CeO_2_ nanorods (r-CeO_2_) with the exposed (110) and (100) crystal planes exhibited significantly higher catalytic efficiency and an unheard-before high crotyl alcohol selectivity for selective hydrogenation of crotonaldehyde (Figure 3E). They concluded that surface oxygen vacancies are the active sites for catalyzing crotonaldehyde hydrogenation reaction, which played a key role in controlling the structures of adsorbed C_4_H_6_O by the formed H^−^ from heterolytic H_2_ dissociation and thus determining the crotyl alcohol selectivity.

Besides the crystal plane properties, many studies of CeO_2_ were devoted to clarifying the role of Ce 4*f* electrons under a perspective of electronic structure. It has been demonstrated that surface relaxation and *f* electron localization were believed to be responsible for the observed oxygen vacancy structures and formations. Therefore, understanding Ce 4*f* electrons is important for clearing the distribution of catalytic sites and the catalytic performance of CeO_2_ (Li et al., 2009). The ground state electronic structure of CeO_2_ has been dealt with two approaches due to the controversy about the occupancy of the Ce 4*f* states. Koelling et al. (1983) have pointed out that some covalent bonding is present in CeO_2_ and thus ceria is not completely ionic in early self-consistent field (SCF) band calculations of the bulk CeO_2_. Then Fujimori inferred the presence of partial occupancy of the Ce 4*f* states in CeO_2_ and concluded that the ground state of ceria might be a mixture of two cerium configurations (4*f* ^0^ and 4*f* ^1^). Therein, Ce 4*f* ^0^ has a filled O 2*p* valence band and Ce 4*f* ^1^ shows a partially filled O 2*p*-valence-band (Fujimori, 1984). Skorodumova et al. (2001) have reported the DFT calculations of bulk CeO_2_ and Ce_2_O_3_ in the framework of the full-potential linear muffin-tin orbital (FP-LMTO) method and obtained the best agreement with the experiment for CeO_2_ by treating the cerium 4*f*-functions as part of the valence region. However, Wuilloud et al. (1984) and Marabelli and Wachter (1987) believed that the cerium 4*f* states in CeO_2_ are fully unoccupied and localized. In this case, Ce was treated as tetravalent Ce^4+^ with an unoccupied 4*f*-band (4*f* ^0^) and a completely filled O 2*p*-band. Under the assumption that the Ce 4*f* orbitals is unoccupied, Hill and Catlow (1993) and Gennard et al. (1999) have neglected completely the Ce 4*f* basis functions and discovered that the bulk properties of CeO_2_ were able to be well described even without the Ce 4*f* electrons by using a minimal basis set on Ce and O and a more extended basis set, respectively.

### 2.2 Defect chemistry of ceria

Typically, CeO_2_ was used as an oxygen buffer in the three-way catalyst, as the quick and reversible redox between Ce^4+^ and Ce^3+^ ensures fast transfer of gaseous oxygen molecules on the solid CeO_2_ surface. In most studies, the excellent catalytic activity has been directly ascribed to its ability to store and release oxygen, i.e., the oxygen storage capacity (OSC). While the OSC of CeO_2_ is associated with the efficient supply of lattice oxygen at reaction sites determined by oxygen vacancy formation. Therefore, understanding the vacancy engineering as well as the unique defect thermodynamics of CeO_2_ at the atomic level is essential to guide the design of CeO_2_-based catalysts. Defects in the crystal structure are the destruction of the symmetry in the perfectly periodic lattice, which are caused by the displacement of atoms from lattice positions. According to the dimensionality of the defects, the CeO_2_ defect can be categorized into point defects (such as oxygen defects and cerium defects) (Esch et al., 2005), line defects (including dislocation defects) (Cao et al., 2022) and plane defects (containing grain boundary defects) (Hojo et al., 2010).

**FIGURE 4:**
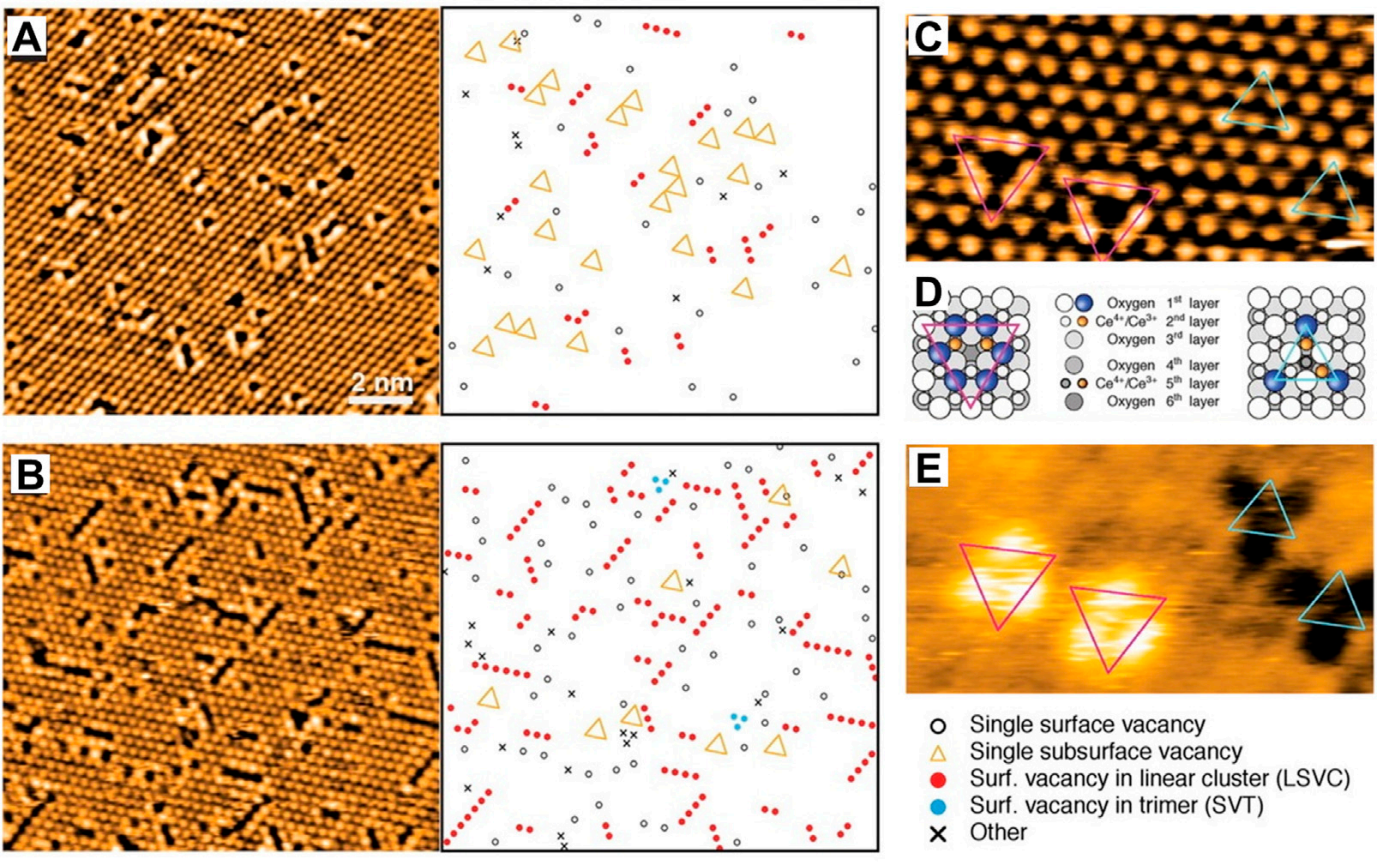
Defect formation on the CeO_2_ (111) surface: **(A,B)** STM images of the CeO_2_ (111) surface under different reduction degree and corresponding representations of the observed defects. **(C–E)** Filled-state **(C)** and empty-state **(E)** STM images of single vacancies, and related structural models **(D)** (magenta triangles mean the surface vacancy and cyan triangles represent the subsurface vacancy). Reproduced with permission (Esch et al., 2005). Copyright 2005, Science.

The point defect refers to the vacancies including oxygen and cerium. Among them, oxygen defects have been widely studied due to their simple structure and extensive applications in catalysis. The oxygen defects of CeO_2_ can be simply divided into intrinsic defects and doped defects according to their origin. Generally, intrinsic defects occur with thermal disorderliness in a crystal following the reductive conversion of Ce^4+^ to Ce^3+^ or the migration of lattice oxygen (Wu et al., 2010; Zacherle et al., 2013; Yang et al., 2021). Doped defects are caused by replacement for normal atoms/particles or occupation of the interstitial site in normal nodes when introducing heteroatoms/particles. The defect form (oxygen or cerium defects) and concentration can be tuned by changing the valence states of doped ions. A variety of defects can be formed on CeO_2_ crystal surface with the valence state changing of Ce ion, including point defects, as well as line-type and triangular-type defect clusters, resulting from multiple point defects (Fukui et al., 2002). Esch et al. (2005) have systematically studied the local structure of the surface and subsurface oxygen vacancies on the CeO_2_ (111) facet by using high-resolution scanning tunneling microscopy (STM) and DFT calculations. They found that single vacancies prevail on the slightly reduced surface and linear surface oxygen vacancy clusters appear and grow upon further reduction (Figures 4A,B). On the slightly reduced surface, single vacancies are distinguished to be surface oxygen vacancies and subsurface oxygen vacancies. Upon further reduction, the linear surface oxygen vacancy clusters appear in three different orientations, reflecting the threefold symmetry of the substrate (Figures 4C–E). Specifically, they suggested that electrons localized on cerium ions by releasing oxygen and then clusters of more than two vacancies exclusively expose these reduced cerium ions, primarily by including subsurface vacancies, which therefore play a crucial role in the process of vacancy cluster formation.

**FIGURE 5:**
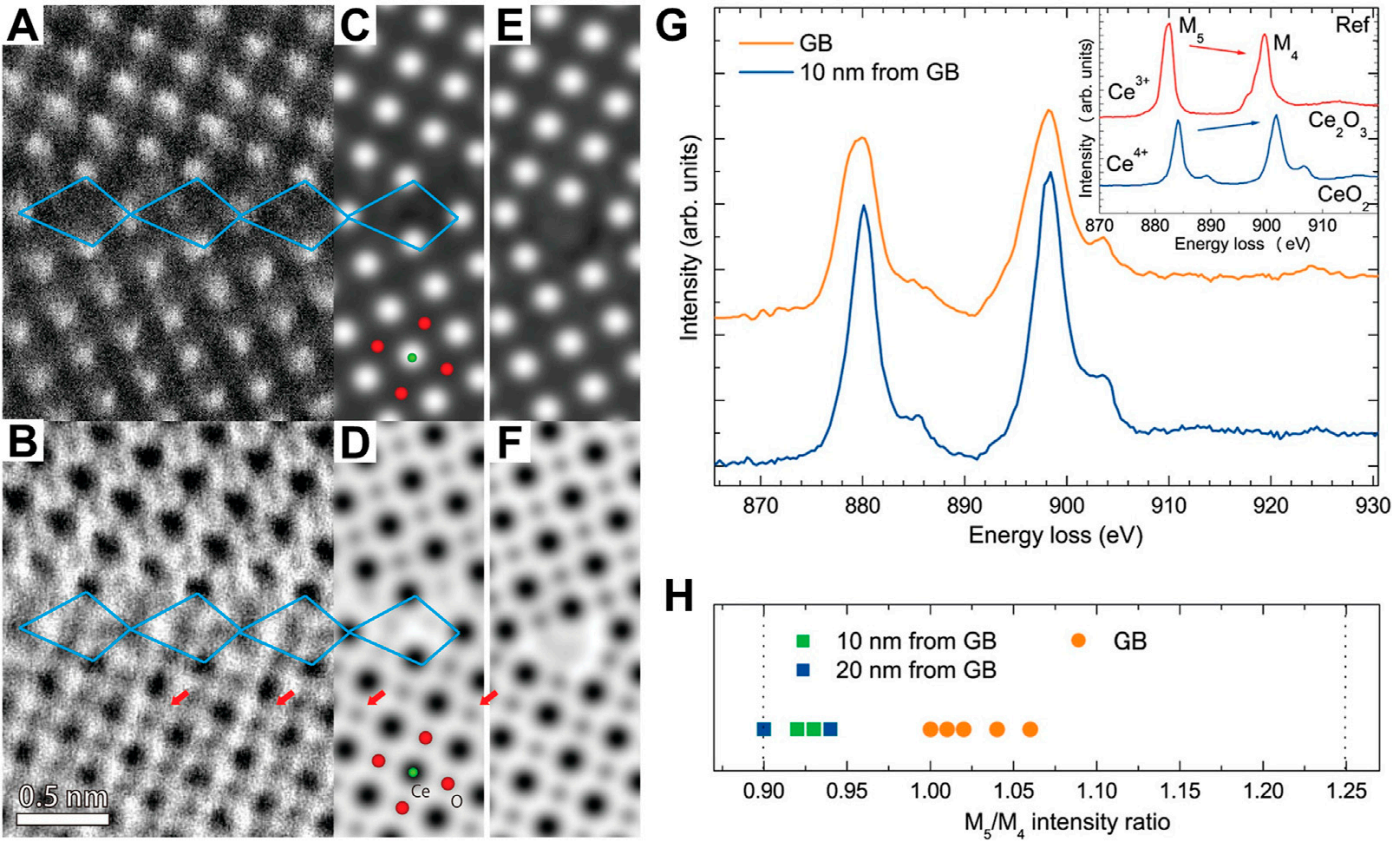
Atomic structure of a CeO_2_ grain boundary: **(A,B)** HAADF **(A)** and ABF **(B)** images of a (210)Σ5 grain boundary in a CeO_2_ thin film. **(C–F)** Simulated HAADF and ABF images of the non-stoichiometric **(C,D)** and stoichiometric **(E,F)** grain boundary model structure. **(G,H)** Typical Ce M_4,5_-edge EELS spectra taken from the grain boundary and interior region and variation of the M_5_/M_4_ intensity ratio at the two different regions. Reproduced with permission (Hojo et al., 2010), Copyright 2010, American Chemical Society.

As introduced above, the periodically linear permutation of the point defects can be regarded as a line defect, which derives from the periodically crystal destruction in a line area (Hojo et al., 2011). A plane defect is produced by a region deviating from periodicity in a crystal, for example, faulting and grain boundary. Among, the grain boundary-derived plane defect is the most widespread. Specific grain boundary structures and non-stoichiometry are produced at grain boundaries in oxides because of the structural discontinuity and higher atom energy. These characteristics have significant impacts on the mechanical and electrical properties such as electronic conductivities and oxygen ionization. In order to understand the macroscopic electrical and chemical properties of CeO_2_, Hojo et al. have investigated the atomic and electronic structures of a (210)Σ5 CeO_2_ grain boundary by using scanning transmission electron microscopy (STEM) and theoretical calculations (Hojo et al., 2010). They observed that the grain boundary consists of repeating structural units, which were marked by quadrilaterals in Figures 5A–F. The structural units repeat over stretches of an interface length of 10–20 nm accompanied with steps in between these stretches. Although the atomic number for oxygen is too small to clearly observe, oxygen columns were directly identified in the annular bright-field (ABF) image (Figures 5D,F). The non-stoichiometric and stoichiometric grain boundaries were also given as shown in the simulated high-angle annular dark-field (HAADF) images of Figures 5C,E, respectively. Further simulations of the model structures revealed that the stable conditions of non-stoichiometric and stoichiometric grain boundary are the reducing conditions of μ_O_ < −2.5 eV and a higher μ_O_ (i.e., oxidizing conditions), respectively. Accordingly, they inferred that the reducing atmosphere benefits to the preferential formation of the non-stoichiometric grain boundary. By the electron energy-loss spectroscopy (EELS) measurements, they confirmed the presence of oxygen vacancies at the grain boundary and the reduction tendency of the cerium ions at the grain boundary region due to the nearby oxygen vacancies (Figures 5G,H). This work revealed that oxygen non-stoichiometry is significant for the stable grain boundary structure of ceria by sufficient experimental evidence, which paves the way for a comprehensive understanding of grain boundaries through atomic scale determination of atom and defect locations.

**FIGURE 6:**
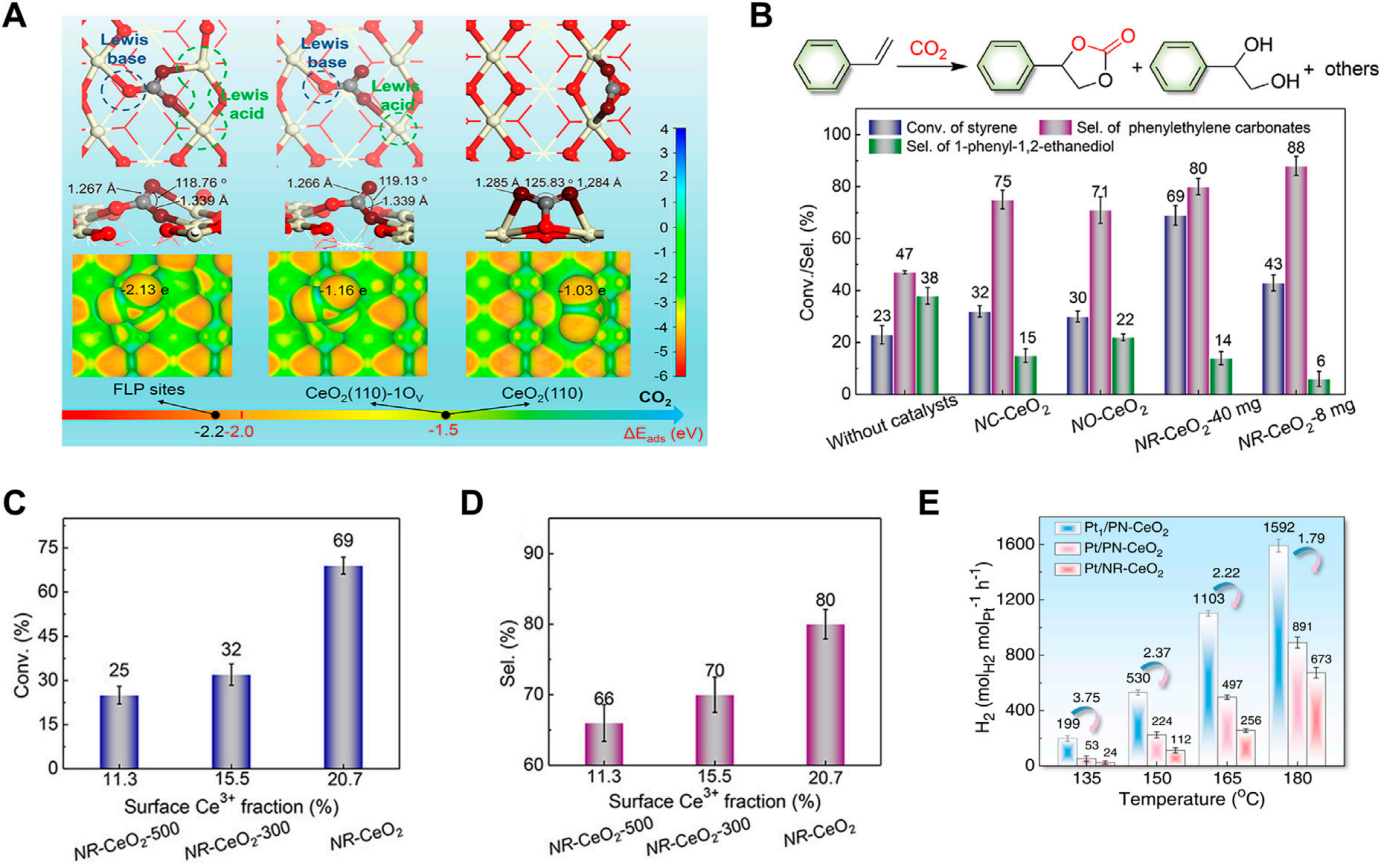
Frustrated Lewis acid-base pairs (FLPs) of CeO_2_: **(A)** FLPs construction and adsorption configurations of CO_2_ on CeO_2_ (110) with different surface properties. **(B–D)** Catalytic performance of various CeO_2_ catalysts for CO_2_ activation for cycloaddition (reaction conditions: styrene (4 mmol), *t*-butylhydroperoxide (0.65 ml, 70 wt% aqueous solution), tetrabutylammonium bromide (40 mg), CeO_2_ (40/8 mg), 80°C, 2 MPa CO_2_, 14 h). Reproduced with permission (Zhang et al., 2019), Copyright 2019, American Chemical Society. **(E)** H_2_ generation rates at different reaction temperatures catalyzed by Pt anchored CeO_2_ catalyst with different Pt sizes or CeO_2_ defect concentration. Reproduced with permission (Zhang et al., 2022), Copyright 2022, Springer Nature.

Besides, the defect engineering is generally used to construct a frustrated-Lewis-pair (FLP) catalyst. As discussed above, CeO_2_ (110) exhibits the best reducible and active with the lowest vacancy-formation energy. Similarly, CeO_2_ (110) presents the highest possibility for FLPs construction (Huang, 2016; Zhang et al., 2017). Many studies have proposed that FLPs constructed on defective ceria possess high activities for many small-molecule activation (such as, H_2_, NO, CO_2_) (Mo et al., 2015; Stephan and Erker, 2015; Huang et al., 2018; Li J. et al., 2021; Li et al., 2022; Zhang et al., 2022; Zou et al., 2022). Qu’s group have explored the adsorption and activation of CO_2_ on CeO_2_ (110) surface by DFT calculations and found that CO_2_ can be easily activated on defective CeO_2_ and further activated on FLPs because of the more negatively charged oxygen of CO_2_, when compared to CO_2_ on an ideal CeO_2_ (110) surface (Figure 6A) (Zhang et al., 2019). This group have also prepared a porous nanorods of ceria (*PN*-CeO_2_) with mainly exposed (110) and (100) facets and constructed the solid FLPs by controlling surface defects of *PN*-CeO_2_ for efficient hydrogenation (Zhang et al., 2017). They concluded that the high concentration of surface defects is important for constructing the FLP sites and improving their capability for H_2_ activation. As shown in Figure 6A, FLPs are combinations of Lewis acids (the reduced surface cerium atoms) and Lewis bases (the “fixed” surface lattice oxygen) that are sterically prevented from interaction to form Lewis acid-base adjuncts (Zhang et al., 2017; Ma et al., 2018). The surface properties, including electronic structures, defect concentration and spatial distance between Lewis acid and base sites (Ce···O), are critical for construction of a new surface Lewis acidic center (Huang et al., 2018; Ma et al., 2018). Meanwhile, the acidity and basicity of Lewis sites and the spatial distance between Lewis acid and base sites are correlation with the catalytic activity (Stephan and Erker, 2015). There were many works to build the relationship between FLPs and catalytic activities. Zhang et al. compared different CeO_2_ crystals, including CeO_2_ nanorods (*NR*-CeO_2_) with FLPs, and CeO_2_ cubes (*NC*-CeO_2_) and octahedra (*NO*-CeO_2_) without FLPs, to investigate their catalytic performance for cyclic carbonate production from a tandem transformation of olefins and CO_2_. These CeO_2_ catalysts have the similar surface Ce^3+^ fractions of ∼20.8% and surface oxygen vacancy percentages of ∼24%. As expected, *NR*-CeO_2_ with FLPs delivered the highest styrene conversion and selectivity for cyclic carbonates (Figure 6B). To eliminate the influence of morphology on the formation of FLPs, *NR*-CeO_2_ with less surface defect percentages were prepared by calcination at 300°C and 500°C in air. The best activity and highest selectivity were also achieved by the defect-enriched *NR*-CeO_2_ sample (Figures 6C,D). Furthermore, they synthesized a porous nanorods of CeO_2_ (*PN*-CeO_2_) with a high surface Ce^3+^ fraction and obtained an improved selectivity of 94% for cyclic carbonates. Subsequently, Zhang et al. have designed a single-atom Pt anchored *PN*-CeO_2_ catalyst (Pt_1_/*PN*-CeO_2_) with dual-active sites of Pt single-atoms and FLPs on *PN*-CeO_2_ to ensure the effective activation of both CH_3_OH and H_2_O for high efficient H_2_ generation at low temperatures (Figure 6E) (Zhang et al., 2022).

## 3 Applications in energy storage and conversion

### 3.1 Photocatalytic applications

Photocatalysis is a green chemical pathway with important application prospects in the fields of energy conversion and environmental protection, which has the advantages of simple operation, low energy consumption, no secondary pollution, and high efficiency. Besides, photocatalysis is an important solar fuel production technology due to its potential for producing valuable compounds while mitigating carbon dioxide emissions. In this respect, semiconductor photocatalysis has been widely used in CO_2_ reduction (Tran et al., 2022), hydrogen evolution (Dung Van et al., 2021), N_2_ reduction to synthesize ammonia (Han et al., 2021), pollutant degradation (Dai Y. et al., 2018), photolysis of water (Wei et al., 2019), etc. In photocatalysis, semiconductors with a smaller half-band gap are more favorable for the photocatalytic reaction and widely used as photocatalysts, whose electrons (e^−^) are more easily activated and transferred from the valence band to the conduction band. In the various photocatalyst semiconductors, TiO_2_, CdS, ZnO, MoP, g-C_3_N_4_, etc., have been extensively investigated (Zhao et al., 2020). As is well-known, the experimental band gap of O 2*p*→Ce 4*f* transition is only about 3.2 eV (Corma et al., 2004). On account of its high capacity of store and release oxygen and great chemical stability, CeO_2_ is becoming a great promising candidate (Yang et al., 2019). For example, Ji et al. (2008) synthesized ordered mesoporous CeO_2_ nano-crystalline and obtained a high photocatalytic activity for the decomposition of the azo dye acid orange under visible light illumination, which greatly outperformed TiO_2_ P25. As demonstrated by Arul et al. (2015), the Fe-doped CeO2 hierarchically porous nanostructured showed a great photocatalytic activity for methylene blue degradation under UV–visible light illumination. In this section, the applications in photocatalytic CO_2_ conversion and hydrogen evolution reaction of CeO_2_-based photocatalysts are overviewed.

#### 3.1.1 CO_2_ conversion

In photocatalytic CO_2_ conversion, the regulation of structure and the binding site location plays an important role in improving conversion efficiency. Oxides of rare Earth metals not only exhibit an excellent capability during the CO_2_ adsorption process, but also present a high charge separation efficiency *via* the addition of surface oxygen vacancies (Muhammad et al., 2020). Hence, rare Earth metals have been considered as the highly viable options for photocatalytic CO_2_ conversion. Ceria was widely studied due to its high chemical stability and outstanding oxygen storage-and-release capability. Besides above mentioned advantages, Fiorenza and co-workers have further emphasized the photocatalytic capability of ceria. They pointed out that the combination of light and temperature can greatly enhance the performance of ceria photocatalysis (Fiorenza et al., 2022). Despite the intrinsic ability of pristine CeO_2_, a higher CO_2_ conversion performance is desired by the optimization of the conduction band (CB) position and quick electron-hole recombination. To achieve high activity and product selectivity in photocatalytic CO_2_ conversion, numerous catalysts modified techniques, such as metal or non-metal doping (Wang M. et al., 2022), building heterojunction (Hamidah Abdullah et al., 2017), and oxygen vacancy engineering (Hezam et al., 2020), have been reported, which benefit to promote CO_2_ molecule activation and adsorption. We mainly summarized the improved CeO_2_-based photocatalysts through element doping, heterojunction building and vacancy engineering in this section.

**FIGURE 7:**
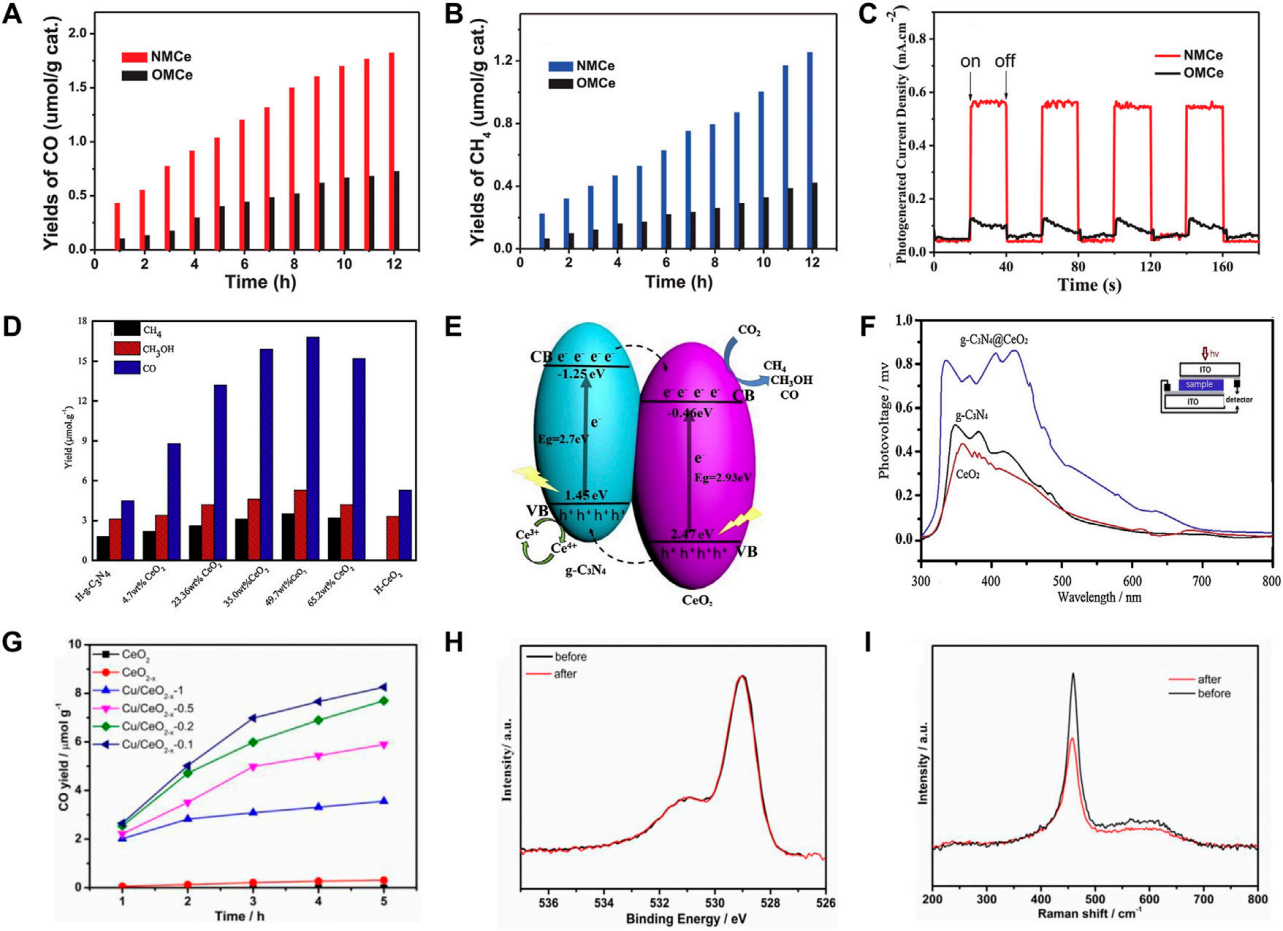
Design of CeO_2_-based photocatalysts for photocatalytic CO_2_ conversion: **(A–C)** A nitrogen-doped mesoporous CeO_2_ (NMCe) as an efficient visible-light-driven catalyst for CO_2_ photoreduction. Reproduced with permission (Shen et al., 2020), Copyright 2020, Elsevier. **(D–F)** Photocatalytic activity and reaction mechanism analysis of hollow heterostructured g-C_3_N_4_@CeO_2_ photocatalysts. Reproduced with permission (Liang et al., 2019). Copyright 2019, Elsevier. **(G–I)** Producing oxygen vacancy with high stability in CeO_2-*x*_ by introducing Cu. Reproduced with permission (Wang M. et al., 2019). Copyright 2019, American Chemical Society.

Elements doping has impacts on the electronic structures of the semiconductor photocatalyst, particularly the bandgap. Transition metal are the common dopant elements for optical and photoelectrochemical semiconductor modification, among which the most widely used include Fe, Ni, Cr, Ag, and so on (Wang Y. et al., 2019; Prajapati et al., 2022). Through the dispersion of Ag particles on CeO_2_ surfaces, Cai and co-authors have investigated the impact of surface plasmon effect on photocatalytic CO_2_ conversion efficiency. It is noteworthy that the Ag-CeO_2_ photocatalyst, which was created *via* a straightforward solvent-based method, can simultaneously produce CH_4_ and CH_3_OH after 6 h of visible light irradiation, giving 100 and 35 mol·g^−1^ h^−1^ of CH_4_ and CH_3_OH, respectively (Cai et al., 2018). Furthermore, in numerous studies, non-metal doping has been mentioned as a viable method to improve photocatalytic activity. Non-metal-loaded elements that could widen the absorption zone to absorb visible light include nitrogen (N) (Shen et al., 2020), sulfur (S) (Wang et al., 2021), and phosphate (P) (Li W. et al., 2021). Excellent CO_2_ reduction was demonstrated by nitrogen-doped CeO_2_, with CO and CH_4_ yields of 1.83 and 1.25 μmol·g^−1^, respectively, being 2.5 times higher than those of nanocasted ordered mesoporous CeO_2_ (OMCe) (Figures 7A–C) (Shen et al., 2020).

The photocatalytic efficiency of pure CeO_2_ as a photocatalyst is insufficient. Heterojunction of CeO_2_ and other nanomaterials is able to overcome the limitations of single component and lead to the synergistic effect, which has been considered as one of the most promising structures to improve its photocatalytic activity. Heterojunction possesses a great benefit in separating photoinduced (e)—(h+) pairs and takes full advantage of the individual functional properties of each component (Zhang W. et al., 2020). Dai and co-workers prepared the CeO_2_/Bi_2_MoO_6_ heterostructured microspheres with different CeO_2_ contents *via* a facile solvothermal route. The heterojunction of CeO_2_/Bi_2_MoO_6_ nanocomposite showed a high specific surface area and a significantly enhanced response to visible light, which is conductive to improve the charge carrier separation and transfer efficiency. As a result, 5% CeO_2_-Bi_2_MoO_6_ with the best activity in photocatalytic CO_2_ reduction toward the generation of CH_3_OH and C_2_H_5_OH realized the yields of 32.5 and 25.9 μmol·g_cat_^−1^ for CH_3_OH and C_2_H_5_OH, respectively (Dai W. et al., 2018). Layered graphitic carbon compound (g-C_3_N_4_) exhibits wonderful photocatalytic activity due to its high reducibility and visual lightweight absorption, but the low specific area and speedy charge recombination in pristine g-C_3_N_4_ have prevented its practical use (Bian et al., 2018). Liang et al. (2019) designed a hollow g-C_3_N_4_@CeO_2_ heterostructure photocatalyst with abundant oxygen vacancies to enhance light utilization and catalytic activities. Because of the unique structure, the synergetic effect and oxygen vacancies, g-C_3_N_4_@CeO_2_ makes multiple reflections of light in the cavity and contributes greatly to the enhanced CO_2_ adsorption capability, thus achieving a much earlier CH_4_ generating and a higher CH_4_ concentration in comparison to that of the pristine g-C_3_N_4_ and CeO_2_. The g-C_3_N_4_@CeO_2_ with a CeO_2_ loading of 49.7 wt% showed the best CO_2_ photoreduction performance with the yields of CH_3_OH (5.2 μmol·g^−1^), CO (16.8 μmol·g^−1^) and CH_4_ (3.5 μmol·g^−1^) (Figure 7D). Further mechanism analysis deduced that the photogenerated electrons in the CB of the g-C_3_N_4_ are able to migrate to the CB of the CeO_2_ while the holes generated in the CeO_2_ will transfer to the valence band (VB) of the g-C_3_N_4_ for the g-C_3_N_4_@CeO_2_ composites (Figure 7E). Such interfacial electron transfer contributes greatly to the separation efficiency of electron-hole pairs resulting and thus the accumulated electron-hole pairs on the CeO_2_ surface promote the generation of CH_4_, which was demonstrated by the obvious surface photovoltage spectroscopy (SPS) signal in visible light region of g-C_3_N_4_@CeO_2_ (Figure 7F). This work provides a novel approach to produce g-C_3_N_4_-based photocatalysts in the absence of noble metal for the high efficiency CO_2_ photocatalytic reduction and clear the charge transfer and catalytic mechanism from the perspectives of structural design and atomic-level regulation.

The poor photocatalytic CO_2_ performances on pure CeO_2_ are mainly due to its wide band gap and low light absorption (Xie et al., 2017). Therefore, oxygen vacancy introduction in the CeO_2_ nanocrystal structure also has attracted strong interest, which can enhance visible-light absorption ability. Introducing oxygen vacancies can make CO_2_ molecules more easily adsorbed and activated on the photocatalyst surface due to the abilities of providing active sites and increasing the CO_2_ adsorption energy. Furthermore, the defect energy level generated by oxygen vacancies is supposed to promote the separation and suppress the recombination of electron-hole and change the transfer path of carriers (Wang et al., 2020). Lai et al. (2022) have introduced surface oxygen vacancies by preparing porous iron-doped ceria, which significantly improved the light absorption performance of ceria in the application of photocatalytic CO_2_ conversion. In spite of introducing oxygen vacancies is able to significantly enhance the CO_2_ photoreduction performance of CeO_2_, its activity would decrease because the stability of the generated oxygen vacancies is insufficient with gradually filling or losing during the photoreduction process. In order to achieve and maintain a high CO_2_ reductive activity of CeO_2_, Wang and co-workers introduce Cu into CeO_2_ to generate and stabilize oxygen vacancies (CeO_2–*x*_) (Wang M. et al., 2019). They found that the Cu-introduced sample show a lower photoluminescence intensity, indicating the extremely enhanced charge transfer between CeO_2_ and Cu species and the effectively inhibited electron-hole recombination, thus leading to a prolonged carriers’ lifetime and stabilized oxygen vacancies. By further catalytic mechanism analysis, they proposed the possible reaction mechanism of photocatalytic CO_2_ conversion on the Cu/CeO_2-*x*_ catalysts and confirmed that the introduction of Cu alters the configurations of the adsorbed CO_2_ on CeO_2–*x*_. Therein, Cu/CeO_2-*x*_-0.1 presented the best photocatalytic performance with a CO yield of 8.25 μmol·g^−1^ during 5 h of Xe-light irradiation and excellent chemical stability (Figures 7G–I).

#### 3.1.2 Photocatalytic hydrogen evolution reaction

Because of the concerns on the sustainability of fossil fuels, photocatalysis is always research focus to create effective, sustainable, and varied energy storage technologies. H_2_ is considered as a promising renewable energy source due to its high energy density of 143 kJ·g^−1^ and the advantages of low emission and no pollution (Xu and Xu, 2015). For a long time, most of the H_2_ is produced by hydrocarbon steam reforming or coal gasification, which are high energy-consuming (Zhao et al., 2020). Currently, the photocatalytic hydrogen evolution reaction (HER) has been considered as a prospective approach to produce H_2_ by an environment-friendly way and attracted lots of research. However, HER is a multielectron, endothermic uphill reaction that requires a high positive Gibb’s free energy (Sultana et al., 2021). Generally, 2.458 eV energy is required to split one water molecule to generate one hydrogen molecule, thus a highly active photocatalyst that possesses capability of decreasing the energy barrier is necessary. Numerous semiconductor photocatalysts with a narrow bandgap and imperative photoredox behavior have been applied in the photolytic HER. Recently, on account of the easy conversion between Ce^3+^ and Ce^4+^ and abundant oxygen vacancies, CeO_2_ has been used in the photocatalytic HER. Dong et al. have synthesized the CeO_2_ nanorods and found that the pure CeO_2_ presented a favorable photocatalytic activity with a high H_2_ production rate of ∼25.10 μmol·g^−1^ (after solar light irradiation for 5 h) (Dong et al., 2018). Moreover, a variety of CeO_2_-based nanostructures, such as Au/CeO_2_ (Primo et al., 2012), ZnO/CeO_2_ (Zeng et al., 2014), CeO_2_/g-C_3_N_4_ (Zou et al., 2017), etc., were reported to strengthen the photocatalytic activity of HER. In this section, we overviewed the mechanism of photocatalytic water splitting for H_2_ production on CeO_2_-based catalysts. Meanwhile, applications of various CeO_2_-based nanostructures in photocatalytic HER were summarized from pristine CeO_2_ to element-doped CeO_2_ and CeO_2_-based heterostructure.

**FIGURE 8:**
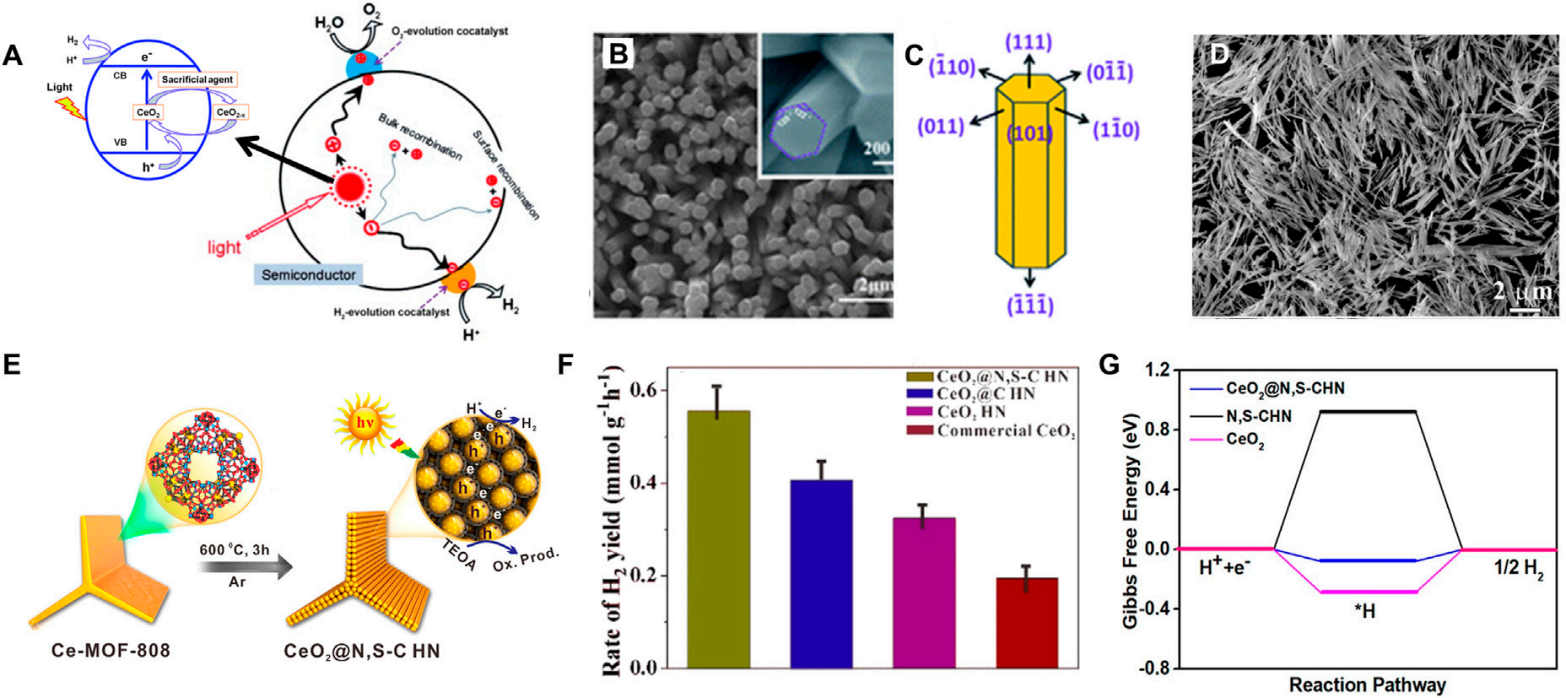
Applications of CeO_2_-based nanostructures for photocatalytic hydrogen evolution reaction: **(A)** Processes involved in CeO_2_-based photocatalytic water splitting reaction. Reproduced with permission (Lu et al., 2011; Xu and Xu, 2015) Copyright 2015, Elsevier. **(B,C)** The oriented hexagonal CeO_2_ nanorods with (110) as the predominantly exposed planes. Reproduced with permission (Lu et al., 2011). Copyright 2011, Royal Society of Chemistry. **(D–G)** Combining N,S-codoped C and CeO_2_ in a 3D hinge-like structure for efficient photocatalytic hydrogen evolution. Reproduced with permission (Hao et al., 2020). Copyright 2020, American Chemical Society.

As introduced by previous reports, water splitting over ceria mainly includes water hydroxylation and H_2_ formation. As shown in Figure 8A, water molecule first adsorbs by the oxygen atom on the top of the cerium atom of CeO_2_ (111) and occurs dissociation near the oxygen vacancy of defect enriched CeO_2_ (111) accompanied by the bonding between one hydrogen atom of water and the surface oxygen atom of CeO_2_. Water dissociation into hydroxyl occurs, followed by hydroxyl decomposition and H_2_ liberation through an asymmetric process. Therein, the surface vacancies facilitate the water dissociation step and the process is accompanied by the oxidation of Ce^3+^ to Ce^4+^ (Lu et al., 2011; Chen et al., 2013; Hansen and Wolverton, 2014; Wu et al., 2019). Because different CeO_2_ facets show different oxygen and cerium coordination, many studies have developed CeO_2_ nanostructures with highly active exposed crystal planes in photocatalytic HER. Tong and co-authors prepared the oriented hexagonal CeO_2_ nanorods with (110) as the predominantly exposed planes by a facile electrochemical method (Figures 8B,C) (Lu et al., 2011). Hexagonal CeO_2_ nanorods present an excellent redox capability thus a super photocatalytic activity for HER. The H_2_ evolution rate on CeO_2_ nanorods was about 741 μmol g^−1^ h^−1^ with Na_2_S-Na_2_SO_3_ sacrificial agents, which was much higher than that of the commercial CeO_2_ or CdS. However, the photocatalytic efficiency of pure CeO_2_ is still not desirable on account of the rapid electron-hole recombination and low proportion of surface active sites. Therefore, non-metallic element doped CeO_2_ and CeO_2_-based heterojunction structures have been extensively studied. Hao et al. synthesized a N,S-doped C-encapsulated CeO_2_ hinge-like nanostructure (CeO_2_@N,S-C HN) to improve the separation efficiency of photoinduced electrons-hole pairs and expose more accessible active sites in the photocatalytic reactions (Figures 8D,E) (Hao et al., 2020). The CeO_2_@N,S-C HN showed a mass-normalized rate of H_2_ production of 555 μmol h^−1^·g^−1^, which is remarkably higher than that of CeO_2_@C HN (405 μmol h^−1^·g^−1^), CeO_2_ HN (325 μmol h^−1^·g^−1^), and commercial CeO_2_ (195 μmol h^−1^·g^−1^) (Figure 8F). Moreover, the CeO_2_@N,S-C HN had a long-term stability without visible activity degeneration after four cycles. Through the spin-polarized DFT calculations, they found that the CeO_2_@N,S-C HN with a lower Gibbs free energy for the formation of the intermediate state (H*) of 0.08 eV exhibited the best HER performance in comparison to pristine CeO_2_ (0.30 eV) and N,S-C (0.93 eV) (Figure 8G). They further deduced that the strong interaction between N,S-C HN and CeO_2_ (111) was beneficial to the photogenerated carriers transfer and separation, thereby enhancing the catalytic performance for HER. In order to reduce the bandgap energy and enhance plasmonic characteristics toward the visible-light range, introducing nitrogen dopants (such as substitutional and interstitial N sites) to ceria is often used, which can increase oxygen vacancies and Ce^3+^ active defects. Van Dao et al. (2021) synthesized a nitrogen-doped ceria coupled with nitrogen-doped graphene (3.9% N-CeO_2_/N-Gr), which exhibited better photocatalytic properties than N-CeO_2_ and CeO_2_. In this case, a super HER rate of 3.7 μmol·mg_cat_^−1^·h^−1^ under visible-light irradiation and remarkable durability were realized by the obtained N-CeO_2_/N-Gr photocatalyst. The reduced bandgap energy confirmed by the DFT calculations can be ascribed to the synergistically electronic effects between 3.9% N-CeO_2_ and N-Gr. Besides, the unique structure with a N-Gr shell, N-Gr network and 3.9% N-CeO_2_ core benefits to play the best role for every component. For the N-CeO_2_/N-Gr photocatalyst, the N-CeO_2_ possesses more oxygen vacancies and Ce^3+^ active defects for enhancing plasmonic properties in the visible-light range; and the N-Gr perfectly performs as an electron reservoir to accumulate plasmon-induced electrons traveling from 3.9% N-CeO_2_ and to suppress the recombination of photoinduced electron-hole pairs. The photocatalytic performance of the heterojunctions is strongly influenced by the interfacial contact between the CeO_2_ and the other semiconductors (Xu et al., 2014). Therefore, CeO_2_ is often combined with other photocatalysts/co-catalysts. Shen and co-authors composited CeO_2_ with W_18_O_49_ to prepare the novel Z-scheme heterojunction photocatalyst W_18_O_49_/CeO_2_. The W_18_O_49_/CeO_2_ composite showed a high hydrogen production efficiency of ∼0.2061 mmol·g^−1^·h^−1^, which was about 1.93 times higher than that of the pure CeO_2_. They proved that the Z-scheme heterojunction structure at the contact interface of W_18_O_49_ and CeO_2_ greatly increased the accumulation of photo-generated electrons and the separation efficiency of the charge carriers, thus enhancing the photocatalytic performance for hydrogen evolution.

### 3.2 Electrocatalytic applications

Electrocatalysis as a promising energy conversion technique has attracted extensive attention worldwide, which provides a clean and convenient route to transfer the universal sources into value-added chemicals and storage chemical energy *via* battery systems. In the last decades, remarkable efforts have been devoted to the development of cost-efficient electrocatalysts for related reactions such as hydrogen evolution reaction (HER), hydrogen oxidation reaction (HOR), oxygen evolution reaction (OER), oxygen reduction reaction (ORR), methanol/ethanol oxidation reaction, carbon dioxide reduction reaction, nitrogen reduction reaction and sulfur reduction reaction. Currently, noble metals (like Pt or Ru) and noble metal oxides (like IrO_2_ or RuO_2_) are the most outstanding electrocatalysts for these catalytic processes. However, the insufficient reserves and high cost of these materials limit their practical uses (Danilovic et al., 2014). Moreover, the catalytic activity and selectivity for targeted products of these reported electrocatalysts still need to be strengthened. CeO_2_ with a series of unique properties as summarized above has been exploited as an effective and promising electrocatalyst or catalyst support in many electrocatalysis systems in recent years (Wang J. et al., 2019). In this section, the applications of CeO_2_-based electrocatalysts in different electrocatalytic reaction of several representative electrochemical systems, including electrolytic water-splitting devices, proton exchange membrane fuel cells, solid oxide fuel cells and lithium-sulfur batteries, are overviewed.

#### 3.2.1 Electrolytic water-splitting devices

**FIGURE 9:**
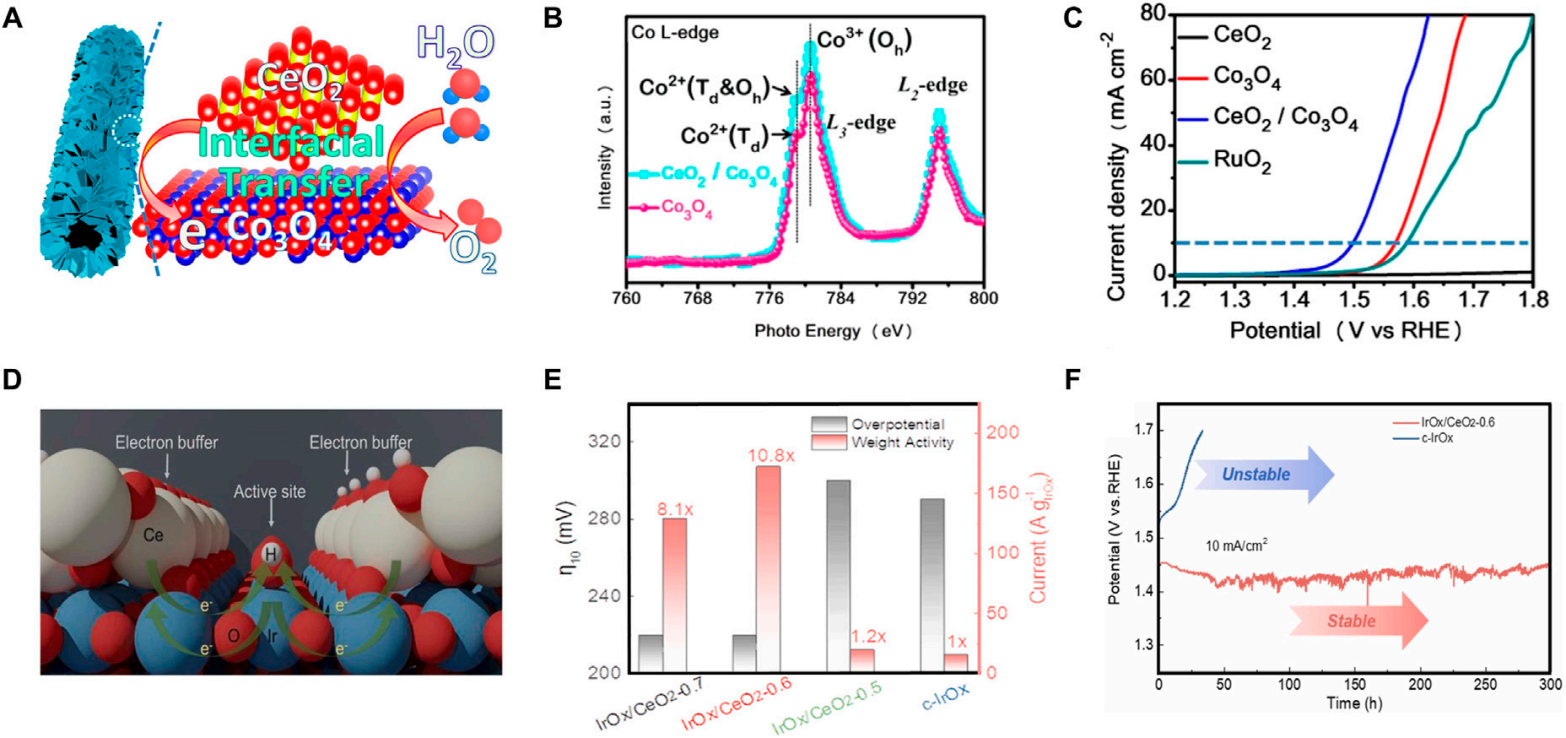
Applications of CeO_2_ in electrolytic water-splitting: **(A–C)** The interaction between catalytically inactive CeO_2_ and spinel structure Co_3_O_4_ **(A)**, and the characteristics of chemical status **(B)** and electrocatalytic activity **(C)** for the as-made Co_3_O_4_-based catalysts. Reproduced with permission (Qiu et al., 2019). Copyright 2019, American Chemical Society. **(D–F)** Catalysis mechanism of IrO_*x*_/CeO_2_ **(D)** and the electrocatalytic performances **(E,F)**. Reproduced with permission (Gou et al., 2022). Copyright 2022, Elsevier.

Splitting water electrochemically and its reversed process form hydrogen and oxygen cycle for energy storage and energy conversion, which involve four critical half-cell reactions, i.e., the HER and oxygen evolution reaction (OER) for energy storage by water electrolysis, and the hydrogen oxidation reaction (HOR) and oxygen reduction reaction (ORR) for energy conversion in fuel cells. Photocatalytic HER is a promising approach to producing H_2_ in an environment-friendly way, CeO_2_-based photocatalysts for HER have been overviewed in the previous section of photocatalytic applications. In this section, we mainly focus on the OER in the water-splitting process. Because of the slow kinetics of the four-electron process, it is generally known that the OER is the bottleneck in the water-splitting processes. In this regard, it is of a great desire to design active OER catalysts that can accelerate O-H bond breaking and O-O bond formation (Gao et al., 2018; Zhang et al., 2021). On account of its superior ionic conductivity and large oxygen storage capacity, CeO_2_ is frequently utilized as a cocatalyst to enhance the charge transfer and energy conversion efficiency of OER catalysts, as well as the OER kinetics (He et al., 2019). The synergistic effect, high surface area, and unique structure of the catalyst all contribute to the enhanced activities (Zhao et al., 2018). Cobalt-based spinel oxides, as one of the promising OER electrocatalysts in alkaline medium, show limited catalytic activity due to the abundant existence of relatively inactive Co^3+^ octahedral coordination (Wang et al., 2016). Qiu and co-workers have built the CeO_2_-induced interfacial Co^2+^ octahedral sites and oxygen vacancies to improve the OER performance of Co_3_O_4_ (Figure 9A) (Qiu et al., 2019). As shown in Figure 9B, the ratio of Co^3+^/Co^2+^ in CeO_2_/Co_3_O_4_ was lower than that of the pristine Co_3_O_4_, accompanied by the increased ratio of Ce^4+^/Ce^3+^ and oxygen vacancies, which was evidenced by X-ray photoelectron spectroscopy (XPS) and Co L-edge X-ray absorption near-edge structure (XANES). As expected, CeO_2_/Co_3_O_4_ interfacial nanotubes show excellent OER performance with an obviously decreased overvoltage of 265 mV at a current density of 10 mA·cm^−2^, which is much lower than those of Co_3_O_4_ (340 mV) and commercially available RuO_2_ catalysts (360 mV) (Figure 9C). Meanwhile, CeO_2_/Co_3_O_4_ interface nanotubes with a Ce/Co ration of 2:20 can achieve an ultrahigh mass activity with a current density of 128.6 A·g^−1^ at a given overpotential of 340 mV and enable an OER Faradaic efficiency of ∼99%. Li et al. (2022) have reported a very different conclusion on the valence of active Co ions by using CeO_2_ nanoparticles anchored Co layered double hydroxide (LDH) as a catalyst. They deemed that the Co^3+^ with strong Lewis acidity helps the binding of OH^−^ and thus benefiting the formation and transformation of oxygen-containing intermediates by forming CoOOH active species (Li Z. et al., 2021). Besides the electrochemical water-splitting in alkaline electrolyzers, the proton exchange membrane water electrolyzers show more promise for practical applications and have benefits for overall water-splitting. However, OER electrocatalysts in acidic conditions are facing great challenges in their longevity on account of the highly oxidizing and corrosively acidic operating environments. Gou and co-workers have prepared the amorphous IrO_*x*_/CeO_2_ nanowire electrocatalysts, featured by nanoscale intimacy and amorphous structure, for water oxidation in 0.5 M H_2_SO_4_, which are providing abundant binary interfaces and favorable kinetics for acidic OER (Gou et al., 2022). They pointed out that CeO_2_ as an electron buffer can not only regulate the adsorption of oxygen intermediates and lowers the activation barrier of OER, but also suppress the over-oxidation and dissolution of iridium (Figure 9D). As a result, IrO_*x*_/CeO_2_ significantly enhanced the OER activity and stability. Iridium/CeO_2_ ratios of 0.6 M (IrO_x_/CeO_2_-0.6) delivered the best electrocatalytic OER performances with a high mass activity of 167 A g_Ir_^−1^ at 1.51 V, a low overpotential of 220 mV at 10 mA·cm^−2^, and a stable performance for 300 h of continuous operation in acid (Figures 9E,F). This work provides a reasonable strategy for constructing acid-resistant OER electrocatalysts.

#### 3.2.2 Proton exchange membrane fuel cells

**FIGURE 10:**
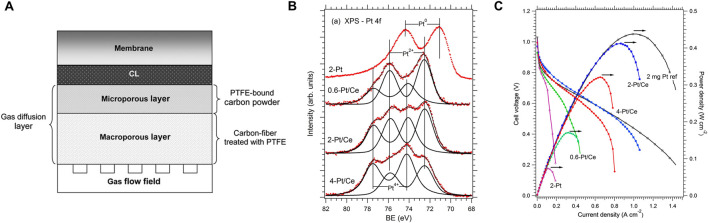
Applications of CeO_2_ in PEMFC: **(A)** Basic structure of a PEMFC. Reproduced with permission (Majlan et al., 2018). Copyright 2018, Elsevier. **(B,C)** High efficient Pt doped cerium oxide thin film as anode for PEMFCs. Reproduced with permission (Fiala et al., 2016). Copyright 2016, Elsevier.

The proton exchange membrane fuel cell (PEMFC) with considerable power density and energy efficiency is one of the most promising candidates for renewable and sustainable energy conversion devices because of its zero CO_2_ emissions, which is widely used as clean energy conversion devices, especially in vehicles and some mobile systems powering. The configuration of PEMFC can be seen in Figure 10A. During the PEMFC working, H_2_ gas at the anode is oxidized to release protons and electrons, then the released electrons generate electricity at the external circuit. The protons, i.e., hydrogen ions, migrate through the polymer electrolyte (proton exchange membrane) to recombine with electrons and oxygen and produce water at the cathode (Majlan et al., 2018). Therein, the catalyst is necessary for accelerating the oxidation of hydrogen gas and reducing oxygen gas to water. The high cost owing to the use of noble metals as catalysts slows the development and commercialization of PEMFCs (Tasic et al., 2009). Therefore, reducing the use of noble metal catalysts and gradually replacing noble metal with a non-noble metal in the anode, is an important direction for the development of PEMFCs.

Fiala and co-workers have reported a novel carbon supported anode catalysts consisting of thin films of ceria with low Pt loadings (Pt^2+^-CeO_2_) (Fiala et al., 2016). They pointed out that the presence of Pt^2+^ is important for enhancing electrocatalytic activity and the stability of Pt^2+^ on ceria enabled the resistance to hydrogenation reduction. Meanwhile, the formation of such stable surface complexes benefits to prevent the degradation of the composite catalysts and improve the durability. To clear the reasonable amount of Pt in the Pt-doped CeO_2_ thin film, the chemical state of Pt was related to the Pt content in the ceria film *via* XPS spectra analysis (Figure 10B). They found that low Pt loadings would give such thin film with Pt species in the +2 state only. However, the excess Pt content would give rise to the appearance of metallic Pt, indicating that the sites for the accommodation of Pt^2+^ in square-planar coordination of O atoms were limited. Remarkably, only small amounts of surface Pt^2+^ can realize a high power density value of 0.41W cm^−2^, which is comparable to the reference commercial catalysts (Figure 10C). When calculated into specific power, this value was 205 kW·g^−1^_(Pt)_, much higher than that of reference commercial catalysts (0.22 kW·g_(Pt)_^−1^).

#### 3.2.3 Solid oxide fuel cells

Solid oxide fuel cells (SOFCs) are a form of fuel cell that consists of a porous anode and cathode separated by a highly dense electrolyte [such as yttria-stabilized zirconia (YSZ) or gadolinium doped ceria (GDC)]. Because of their considerable electrical efficiency, the possibility of using a variety of fuels, and the benign environmental impact, SOFCs have attracted wide attention (Menzler et al., 2010). Although many key materials for SOFCs have been developed in the last decades, there are still great challenges in improving durability and decreasing cost (Suntivich et al., 2011). Currently, the researches on SOFCs materials mainly focus on the optimization of anode, cathode and electrolyte. As CeO_2_ can be not only used as catalysts or catalyst supports in electrodes, but also explored as electrolytes with improved ionic conductivity, it was widely used in SOFCs (Mogensen, 1994). For example, non-doping CeO_2_ nanocubes were used as an electrolyte in advanced fuel cells and exhibited outstanding performance (Li et al., 2018). In the case of the CeO_2_-coated NaFeO_2_ proton-conducting electrolyte, the addition of the CeO_2_ shell layer not only increased the number of oxygen vacancies for proton transport but also introduced heterointerface for enhancing ionic boundary conductivity (Xing et al., 2021). This section summarizes the applications of CeO_2_ used as an electrolyte and electrode component in SOFCs.

**FIGURE 11:**
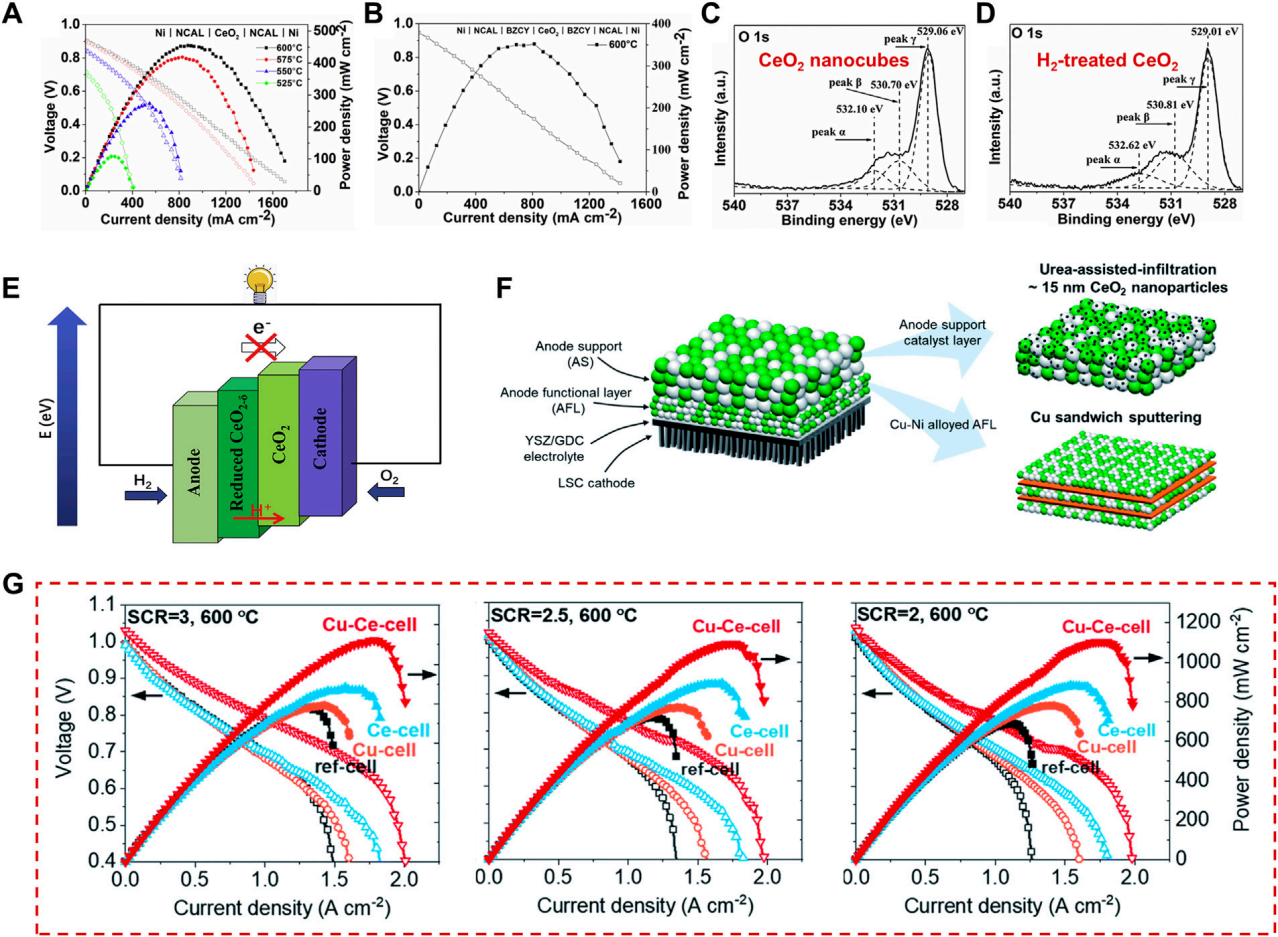
CeO_2_-introducted electrolyte and electrode of SOFCs: **(A–E)** CeO_2_ nanocubes electrolyte and design of the CeO_2_/CeO_2-δ_ heterogeneous interfaces. Reproduced with permission (Li et al., 2018). Copyright 2018, Elsevier. **(F,G)** The configuration and CeO_2_-based catalyst incorporation strategies in a TF-SOFC and the obtained fuel cell performance. Reproduced with permission (Thieu et al., 2022). Copyright 2022, Royal Society of Chemistry.

When used as an electrolyte, ceria is generally doped with other trivalent element (or less commonly bivalent) to realize a significant improvement of the ionic conductivity. Common dopants include calcium (Sudarsan and Moorthy, 2019), yttrium, samarium (Bhabu et al., 2016), and gadolinium (Khan et al., 2019). In comparison to the single-phase electrolytes, doping increases the oxygen vacancy concentration, which dictates ionic conduction. Li and co-workers have synthesized CeO_2_ nanocube with exposed the (100) and (110) active crystal facets and obtained an excellent power density of 406 mW·cm^−2^ at 600 C by using the un-doped CeO_2_ nanocubes as an electrolyte for advanced fuel cell (Figures 11A,B) (Li et al., 2018). To reveal the origin of the excellent fuel cell performance, they calculated the number of oxygen vacancies of the H_2_-treated CeO_2_ (CeO_2-δ_) and CeO_2_ nanocubes from the XPS spectra, which showed an increased amount of oxygen vacancies and a higher Ce^3+^ percentage for the H_2_-treated CeO_2_ (Figures 11C,D). Moreover, a lower activation energy of 0.63 eV was found for CeO_2_ nanocubes-introduced fuel cells, which is superior to that of YSZ (0.91 eV) (Strlckler and Carlson, 1965) and other oxide ion conductors (Rupp and Gauckler, 2006). As confirmed by previous studies, major proton conducting mechanism in oxides is conducted by oxygen vacancies, thus proton conduction is highly related to the concentration of oxygen vacancies. Therefore, they inferred that the surface of the defective CeO_2_ nanocube can enhance proton transport through its abundant oxygen vacancies and its conductivity is governed by interfacial ionic transportation. As shown in Figure 11E, the semiconducting energy band differences between the reduced CeO_2_ (CeO_2-δ_) at the anode and oxidized CeO_2_ at the cathode side form the double layer device, which can achieve effective charge separation and avoid the device short-circuiting. This work demonstrated the CeO_2_/CeO_2-δ_ heterogeneous interfaces with a high ionic conductive path conductor and provided a well electrolyte choice for advanced SOFCs, which widen the selecting range of various electrolyte candidates. Besides, ceria is often combined with other components, for example, transition metal layered oxides (TMLOs), carbonates, oxides (Raza et al., 2011), hydroxides (Hu et al., 2006), sulfates (Liu et al., 2005), and halides (Zhu et al., 2001), etc., to create a better low-temperature SOFCs (LT-SOFCs) (Wang et al., 2008; Benamira et al., 2011; Fan et al., 2013). Xing and co-workers reported the CeO_2_ coated NaFeO_2_ as an electrolyte material for LT-SOFC (Xing et al., 2021). They found that the CeO_2_ shell layer introduces more oxygen vacancies for proton transfer in the obtained electrolyte material. Meanwhile, the heterointerface is in favor of the O^2−^ grain boundary conductivity. As a result, both the open-circuit voltage (OCV) and the power output of the fuel cells were greatly improved. The fuel cell delivered an admirable power output of 727 mW·cm^−2^ at 550°C by using the core cell NaFeO_2_-CeO_2_ composites as an electrolyte.

Another widely explored field in SOFCs developments is ceria-based composite cathodes and anodes, such as Pd@CeO_2_ (Adijanto et al., 2013), Ru-CeO_2_ (Zhan and Barnett, 2005), Ni-CeO_2_ (Lee et al., 2013), and so on. As a high cost of the noble-metal catalysts, Thieu and co-workers prepared the catalyst-modified cells with Cu and CeO_2_ (Cu-Ce-cell), in which Cu was inserted directly near the electrolyte-anode interface and CeO_2_ was incorporated into the anode support to effectively facilitate thermochemical reforming reactions (Figure 11F) (Thieu et al., 2022). As shown in Figure 11G, Cu-Ce-cell exhibited a record high performance with a peak power density of 1,120 mW·cm^−2^ at 600°C. The peak power densities of Cu-Ce-cell did not significantly change with the different steam-to-carbon ratios (SCRs). Furthermore, Cu-Ce-cell showed long-term performances with only slight voltage degradation of ∼2.76% over 250 h under a constant current load of 0.15 A·cm^−2^ by using butane fuel with an SCR of 3 at 600°C.

#### 3.2.4 Sulfur conversion reaction in lithium-sulfur batteries

**FIGURE 12:**
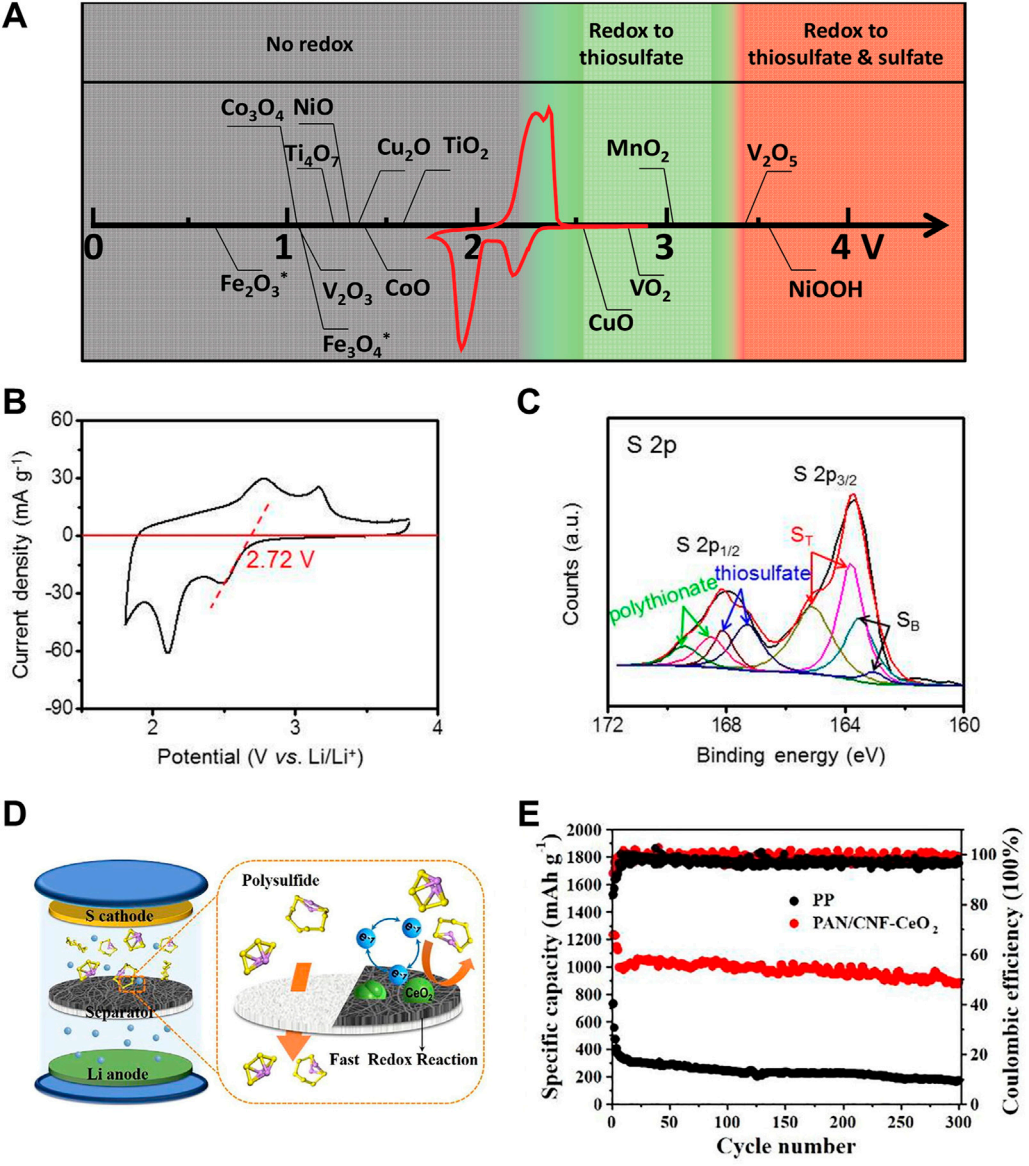
Applications of CeO_2_ in Li-S batteries: **(A)** Chemical reactivity of different metal oxides with LiPSs as a function of redox potential versus Li/Li^+^, superimposed with a typical Li-S cyclic voltammetry curve. Reproduced with permission (Liang et al., 2016). Copyright 2016, Wiley Online Library. **(B)** Chemical reactivity of CeO_2_. **(C)** High-resolution XPS spectrum at S 2*p* region after the adsorption test of Li_2_S_4_ with CeO_2_/MMNC. Reproduced with permission (Ma et al., 2017). Copyright 2017, American Chemical Society. **(D,E)** Catalytic mechanism and performance of a Li-S battery with PAN/CNF-CeO_2_ interlayer. Reproduced with permission (Zhang J. et al., 2020). Copyright 2020, Elsevier.

Lithium-sulfur (Li-S) batteries are regarded as a promising energy storage system for new generation portable electronic devices and electric vehicles due to their high theoretical energy density (2,600 Wh·kg^−1^) and specific capacity (1,675 mAh·g^−1^) as well as the low cost, natural abundance, and environmentally friendly nature of sulfur (Urbonaite et al., 2015). However, the insulating property of sulfur and its discharge products leads to limited reaction kinetics during the redox processes, which results in low utilization of sulfur and insufficient practical specific capacity. Furthermore, in the multistep sulfur reduction reaction, the conversion of the soluble lithium polysulfide intermediates (LiPSs) into insoluble Li_2_S_2_/Li_2_S has a much higher apparent activation energy, which will lead to the accumulation of polysulfides in the liquid electrolyte, a continuous loss of active sulfur from the cathode and the final battery failure. Therefore, introducing electrocatalysts in Li-S cells towards fast sulfur conversions is of great significance for decreasing the activation energy of the precipitation of Li_2_S_2_/Li_2_S solids and improving Li-S battery performances (Liu et al., 2021). Metal oxides, sulfides, nitrides, phosphides, and their heterostructures have been screened in a large number of studies (Yuan et al., 2016; Sun et al., 2017; Zhang M. et al., 2020; Hua et al., 2021; Xia et al., 2021). Liang et al. (2016) have pointed out that only materials with redox potentials in a targeted window can react with polysulfides to form active surface-bound polythionate species. And they deemed that the formed active surface-bound polythionate species have direct correlation with the superior Li-S cell performance (Figure 12A). These metal oxides with redox potentials between 2.4 and 3.05 V (such as VO_2_ and CuO) possess a window that lies just above the redox voltage of soluble polysulfides and thus promotes polythionate formation. A higher redox potential will lead to excessive oxidization of polysulfides, and a lower one shows no redox reaction with polysulfides. CeO_2_ is a polar metal oxide that can chemically adsorb the sulfur species and boost redox reaction through catalysis. Meanwhile, CeO_2_ nanocrystals possess a proper redox potential of 2.72 V vs. Li/Li^+^, which is higher than that of polysulfides (2.10 V vs. Li/Li^+^). Accordingly, the CeO_2_ nanocrystals might oxidize the intermediate polysulfides to thiosulfates and polythionates *via* the surface redox chemistry (Figures 12B,C) (Ma et al., 2017). Therefore, many CeO_2_-based nanostructures have been reported to be used as electrocatalysts to promote Li-S cell performance.

Ma and co-workers demonstrate an advanced sulfur host material prepared by implanting CeO_2_ nanocrystals homogeneously into bimodal micromesoporous nitrogen-rich carbon nanospheres (CeO_2_/MMNC) (Ma et al., 2017). This hybrid with high conductivity and abundant hierarchical pore structures can effectively store and capture sulfur species. Meanwhile, the CeO_2_ can promote the chemical redox reactions of polysulfides, thus significantly enhancing their retentions upon cycling. As a result, CeO_2_/MMNC-S cathodes showed the excellent capacity and rate performance, as well as an ultralong cycle life. Specifically, the cathode with a sulfur mass loading of 1.4 mg·cm^−2^ realized the reversible capacities of 1,066 mAh·g^−1^ at 0.2 C after 200 cycles and 836 mAh· g^−1^ at 1.0 C after 500 cycles, and a high cycle stability of 721 mAh·g^−1^ at 2.0 C after 1,000 cycles with a low capacity decay of 0.024% per cycle. Besides carbon nanospheres, other carbon materials, such as carbon nanotubes (CNTs) and graphene, are often used as supports to disperse the active CeO_2_ nanocrystal (Ma et al., 2017). Yuan et al. have designed a three-dimension porous conductive network of CeO_2_-webbed carbon nanotubes (CeO_2_@CNT) to provide fast electron paths and achieve a good rate capability in the Li-S batteries (Xiao et al., 2018). In order to increase the sulfur utilization, CNT particles with high tap density were applied to realize a uniform melt-diffusion of sulfur by Gueon and co-authors. They further demonstrated microdomain sulfur by coating the CeO_2_ nanoparticles in a CNT with an open pore structure. The open pore structure of CeO_2_/CNTP and the microdomain sulfur enabled fast kinetics in the redox reaction of sulfur, and therefore achieving excellent cycling stability of only 0.044% per cycle for 300 cycles at 2 C and a high capacity of 5.6 mAh·cm^−2^ even at high sulfur loading (Gueon et al., 2020). In addition, CeO_2_ was used as cathode materials alone by constructing phosphorus-modulated porous CeO_2_ (P-CeO_2_) as reported by Tao et al. (2022). The P-CeO_2_ cathode showed a better oxidation-reduction kinetics of LiPSs and a faster Li^+^ diffusion rate in comparison to that of bare CeO_2_. Meanwhile, they have confirmed that the P-CeO_2_ cathode presented stronger adsorption of Li_2_S_6_, higher redox peak current, and earlier precipitation of Li_2_S in comparison to the bare CeO_2_. Therefore, introducing P resulted in an improved initial capacity of 1,027 mA·h·g^−1^ (bare CeO_2_: 895.7 mA·h·g^−1^) at 0.2 C.

Apart from the sulfur host, CeO_2_ has also been used in the separator modification. Generally, the soluble polysulfides can be immobilized in the cathode side by the multifunctional modified interlayer. Cheng et al. have designed a multifunctional separator modified by CeO_2_ decorated graphene (CeO_2_@G) to accelerate polysulfide redox reaction and immobilize polysulfides by strong chemisorption (Cheng et al., 2021). Zhang and co-authors fabricated a functional bilayer separator based on 0D (CeO_2_ nanocrystals)/1D (carbon nanofibers) composite mats (PAN/CNF-CeO_2_) (Zhang J. et al., 2020). By integrating the advantages of highly conductive carbon nanofibers and electrocatalytically active CeO_2_ nanocrystals, the Li-S batteries with the obtained PAN/CNF-CeO_2_ separators showed high S utilization, excellent thermal stability, superior rate performance and enhanced cycling stability (Figure 12D). Specifically, the Li-S cell exhibited an initial reversible capacity of 1,359 mAh·g^−1^ at 0.2 C and a low capacity decay rate of 0.04% per cycle at 0.5 C over 300 cycles (Figure 12E).

## 4 Summary and outlook

In this review, we introduce the electronic properties and defects engineering of CeO_2_-based nanostructures to understand the relationship between catalytic performance and inherent properties. The typical catalytic applications in energy conversion and storage of CeO_2_-based nanostructures have also been demonstrated. Therein, the mechanisms and key component developments of several photocatalytic reactions and representative energy storage cells have also been summarized. With great progress being made in the synthesis of CeO_2_-based nanostructures, there are fascinating new opportunities and challenges for materials scientists. The understanding of CeO_2_ materials has evolved in the last decades from inert supports through cocatalysts and to the catalyst itself (Montini et al., 2016). The development of nanotechnology made it possible to acquire well-controlled nanomaterials in terms of size and morphology, which has improved our understanding on the catalytic performance optimization of CeO_2_-based nanocatalysts. Moreover, there are many theoretical calculations for providing a guideline on the rational design of highly reactive CeO_2_-based catalysts. In applications for energy storage and conversion through photocatalysis and electrocatalysis, CeO_2_ is frequently utilized as a catalyst or a crucial component of catalysts. In conclusion, CeO_2_ is an extremely adaptable and durable catalytic material with surface acid-base characteristics and a structure that can be finely modified by element doping and introducing other compounds. Although many of the studies on CeO_2_-based nanostructures reported so far have shown considerable progress in its catalytic application, more attentions need to be paid to the synthesis, characterization approaches and practical uses. For example, the precise synthesis methods still need to be paid attention to realize the controllable defects concentration and selectively exposed crystal facets. Introducing other elements or components in CeO_2_ to construct composites, heterojunctions and modifications is commonly, which can regulate the electronic structure of the catalyst or optimize the properties of CeO_2_. However, it is also quite necessary to prepare specifically nanostructured oriented ceria-based systems (for example porous structures, core-shell structures, hollow structures, surface acidity, and basicity of Lewis sites, etc.) to realize desired catalytic performance, in addition to above mentioned. Precise synthesis is not only critical for enhancing catalytic performance but also for providing valuable references for our research on catalytic mechanisms. At present, controlled generation of oxygen vacancies and cerium defects is challenging and needs more experimental explorations. The long-term stability of the CeO_2_ nanostructures under extreme conditions and reaction conditions is of potential concern. Especially, oxygen vacancy stabilization is a noteworthy issue. Oxygen vacancies are generally considered as the important active sites, therefore ensuring the similar densities of oxygen vacancies on CeO_2_-based catalysts after cycling test is one of key metrics. The relationship between material inherent properties and catalytic performances should be understood by a simple and experimentally measurable descriptor instead of mere theory calculations. Further basic understanding of burgeoning novel materials and direct confirmation of the effect on catalytic efficiency are conductive to develop a strong understanding of structure-activity interlinkage and guide researchers to design and synthesize extraordinary CeO_2_ nanostructures.
